# An insight into molecular taxonomy of bufonids, microhylids, and dicroglossid frogs: First genetic records from Pakistan

**DOI:** 10.1002/ece3.8134

**Published:** 2021-09-28

**Authors:** Ayesha Akram, Muhammad Rais, Karem Lopez‐Hervas, Rebecca D. Tarvin, Muhammad Saeed, Daniel I. Bolnick, David C. Cannatella

**Affiliations:** ^1^ Herpetology Lab Department of Wildlife Management Pir Mehr Ali Shah Arid Agriculture University Rawalpindi Rawalpindi Pakistan; ^2^ Department of Wetland Ecology Doñana Biological Station Consejo Superior de Investigaciones Científicas Seville Spain; ^3^ Department of Integrative Biology University of California Berkeley Berkeley California USA; ^4^ Museum of Vertebrate Zoology and Department of Integrative Biology University of California Berkeley Berkeley California USA; ^5^ Department of Ecology and Evolutionary Biology University of Connecticut Storrs Connecticut USA; ^6^ Department of Integrative Biology and Biodiversity Center University of Texas Austin Texas USA

**Keywords:** endemism, *Minervarya*, *Nanorana*, new records, phylogeny

## Abstract

The current study was focused on documentation of amphibian assemblage in North Punjab and Islamabad Capital Territory, Pakistan, by using mitochondrial gene sequences of 16S rRNA. Our study entailed 37% of the known amphibian species of the country. We provided a phylogenetic analysis based on 74 newly generated mitochondrial 16S rRNAs from nine species of genus *Microlyla, Duttaphrynus, Allopaa, Nanorana, Sphaerotheca, Minervarya, Hoplobatrachus,* and *Euphlyctis*. We employed the maximum‐likelihood inference and Bayesian analysis to assess the taxonomic status of the samples obtained from Pakistan, with respect to other congeneric species from surrounding regions. Our findings confirmed the taxonomic status of South Asian anuran species *Duttaphrynus stomaticus, Duttaphrynus melanostictus*, *Microhyla nilphamariensis, Allopaa hazarensis*, *Nanorana vicina, Sphaerotheca maskeyi* (synonym*: S. pashchima), Minervarya pierrei, Hoplobatrachus tigerinus,* and *Euphlyctis kalasgramensis* in Pakistan. We have reported new country records of genus *Minervarya* *(* *M*. *pierrei*). *Minervarya pierrei* was previously misidentified as *Fejervarya limnocharis,* due to dearth of genetic information. We provided the first genetic records of our endemic species *N*. *vicina*. The results revealed the taxonomic placement of *N*. *vicina* with respect to its congeners and validated the taxonomic status of *N*. *vicina* from its type locality (Murree) for the first time. The findings of the present study also indicated the paraphyletic relationship of *A.‐ hazarensis* with *Nanorana* species. So, based on our phylogenetic inferences, morphological characters, and habitat preferences, validity of generic status of *A*. *hazarensis* is undecided. As our data were not enough to resolve this issue, we suggest sequencing of additional mitochondrial and nuclear genes in the future studies to get a better resolution. We recommend carrying out extensive surveys throughout the country for proper scientific documentation of amphibians of Pakistan. Many new species, some of them might be endemic to Pakistan, are expected to be discovered, and taxonomic status of other species would be resolved.

## Graphical abstract

The current study was focused to document amphibian assemblage of North Punjab and Islamabad Capital Territory, Pakistan, by using 16S rRNA. Our findings on the taxonomic status of South Asian anuran species confirmed the presence of *Duttaphrynus stomaticus*, *Duttaphrynus melanostictus*, *Microhyla nilphamariensis*, *Sphaerotheca maskeyi* (synonym: *S. pashchima*) *Hoplobatrachus tigerinus*, *Euphlyctis kalasgramensis* and *Allopaa hazarensis*. We have reported new country records of *Minervarya pierrei* and reported the first genetic records of south asian endemic species *Nanorana vicina* from its type locality.

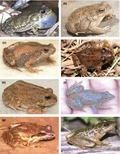

## 1 INTRODUCTION

Identification of species by examining only morphological characters is difficult and may result in misidentifications (Stuart et al., 2006). Modern amphibian taxonomy relies heavily on molecular taxonomy and phylogeny (Frost et al., 2006). In the recent past, integrated taxonomic approaches have been applied successfully to resolve the complications associated with the species identification; see Phuge et al. (2020). In amphibian's taxonomy, many new species are being described worldwide (Frost, 2019) and the discovery of new anuran species is an ongoing activity (Ohler et al., 2009). Despite these new findings and descriptions, the amphibian species in South and South‐East Asia remain underestimated generally due to the presence of homoplasy in morphology of amphibians (Stuart et al., 2006).

Boulenger (1890) provided a detailed description of amphibians of British India (Now Pakistan, India, Myanmar, and Sri Lanka). A total of 348 amphibians have been described so far from the eight countries of South Asia, with significant contribution from India and Sri Lanka (Molur, 2008; Pratihar et al., 2014). The territory of Pakistan, which is influenced by fauna from different geographic directions, is divided zoo geographically into the Palearctic and Oriental regions (Khan, 2006). Pakistan is one of the important territories in Eurasia in respect of past biodiversity dynamics (Jablonski et al., 2020). The amphibians of Pakistan are represented by a heterogeneous assemblage of 24 anuran species belonging to nine genera (*Duttaphrynus, Scutiger, Microhyla, Uperodon, Euphlyctis, Fejervarya, Hoplobatrachus, Allopaa,* and *Sphaerotheca*) distributed over four families Bufonidae, Microhylidae, Megophryidae, and Dicroglossidae (Khan, 2006).

The contributions on diversity and ecology of amphibians of Pakistan include Khan (1976, 2001, 2006), Dubois and Khan (1979), Ohler and Dubois (2006), Ficetola et al. (2010), Yousaf et al. (2010), Tabassum et al. (2011), Rais et al. (2012, 2014), Pratihar et al. (2014), and Akram et al. (2015). Khan (2006) provided a checklist and identification key of anurans of Pakistan. The listing of species and their taxonomic status was based on morphological examination without any genetic confirmation and molecular taxonomy. As there is a vast sampling gap exists in the field of molecular taxonomy, strong uncertainties persist about the taxonomy of many anuran species of Pakistan. Molecular taxonomic studies are crucial to fill this gap. Few phylogenetic studies were recently conducted in Pakistan by Hussain et al. (2020) on genus *Duttaphrynus*, Jablonski et al. (2020) on genus *Microhyla*, Ali et al. (2020) on genus *Euphlyctis*, Jablonski et al. (2021) on genus *Sphaerotheca,* and Hofmann et al. (2021) on genus *Allopaa*. Despite these studies, there is scanty of information and anuran species from Pakistan are still underrepresented. The detailed phylogenetic relationship of anurans within the family Bufonidae, Microhylidae, and Dicroglossidae especially based on the samplings from Pakistan is extremely elusive.

In the present study, we will be focusing on the genetic records of family Bufonidae (genus *Duttaphrynus*), Microhylidae (genus *Microhyla*), and Dicroglossidae (*Allopaa, Nanorana, Minervarya, Sphaerotheca, Hoplobatrachus,* and *Euphlyctis*) from Northern Punjab (Rawalpindi District) and Islamabad Capital Territory, Pakistan. The anuran fauna of this region also include endemic species such as *Nanorana vicina,* which is endemic to South Asia, and *Allopaa hazarensis,* which is endemic to Pakistan. We therefore in this study provided the molecular evidences and assemblage of reported anuran species of North Punjab (Rawalpindi District) and Islamabad Capital Territory, Pakistan.

## 2 MATERIALS AND METHODS

### 2.1 Sampling area

**FIGURE 1:**
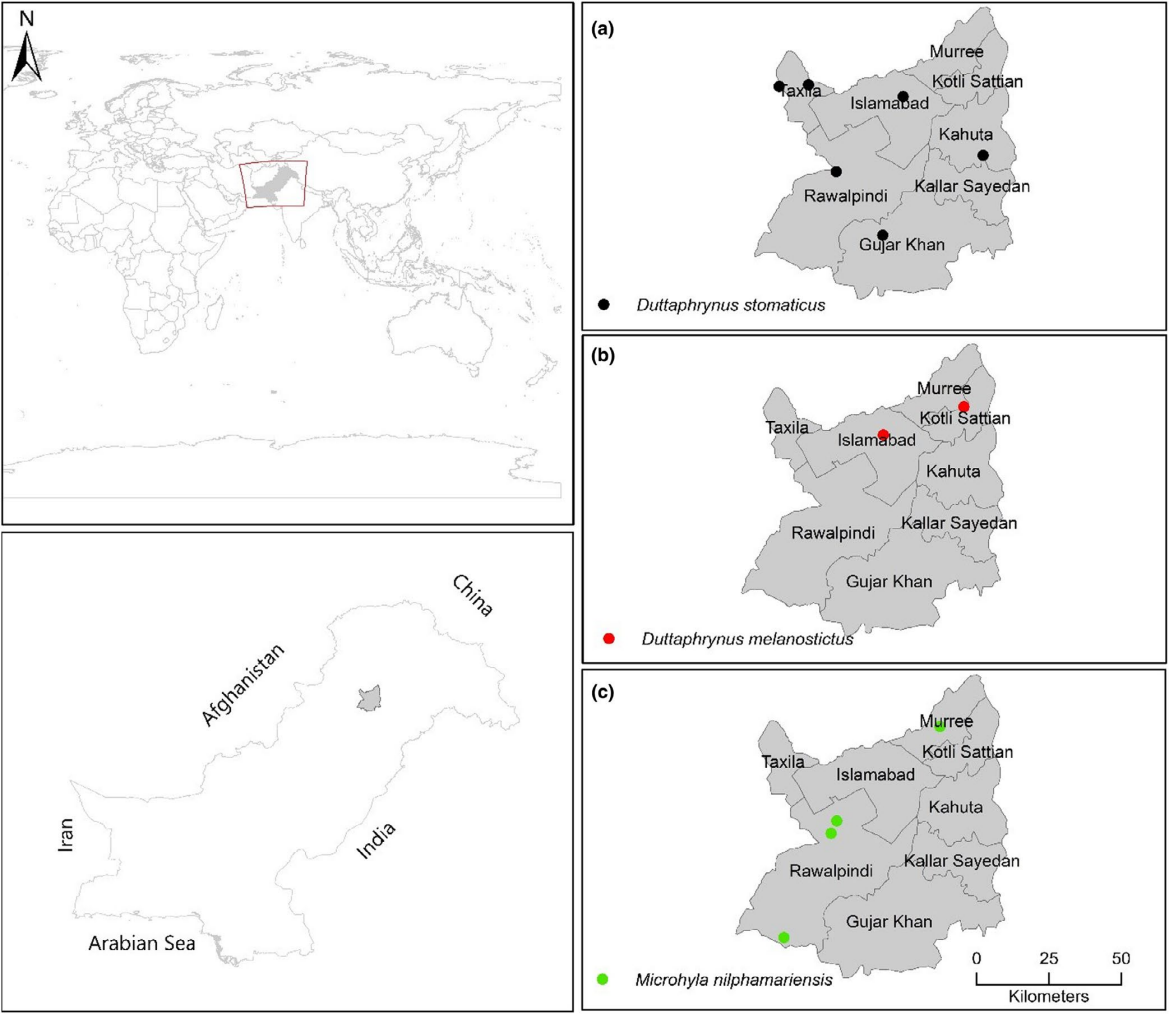
Map of the study area showing sampling locations of genus *Duttaphrynus* and *Microhyla* in Rawalpindi District, Punjab Province and Islamabad Capital Territory, Pakistan

**FIGURE 2:**
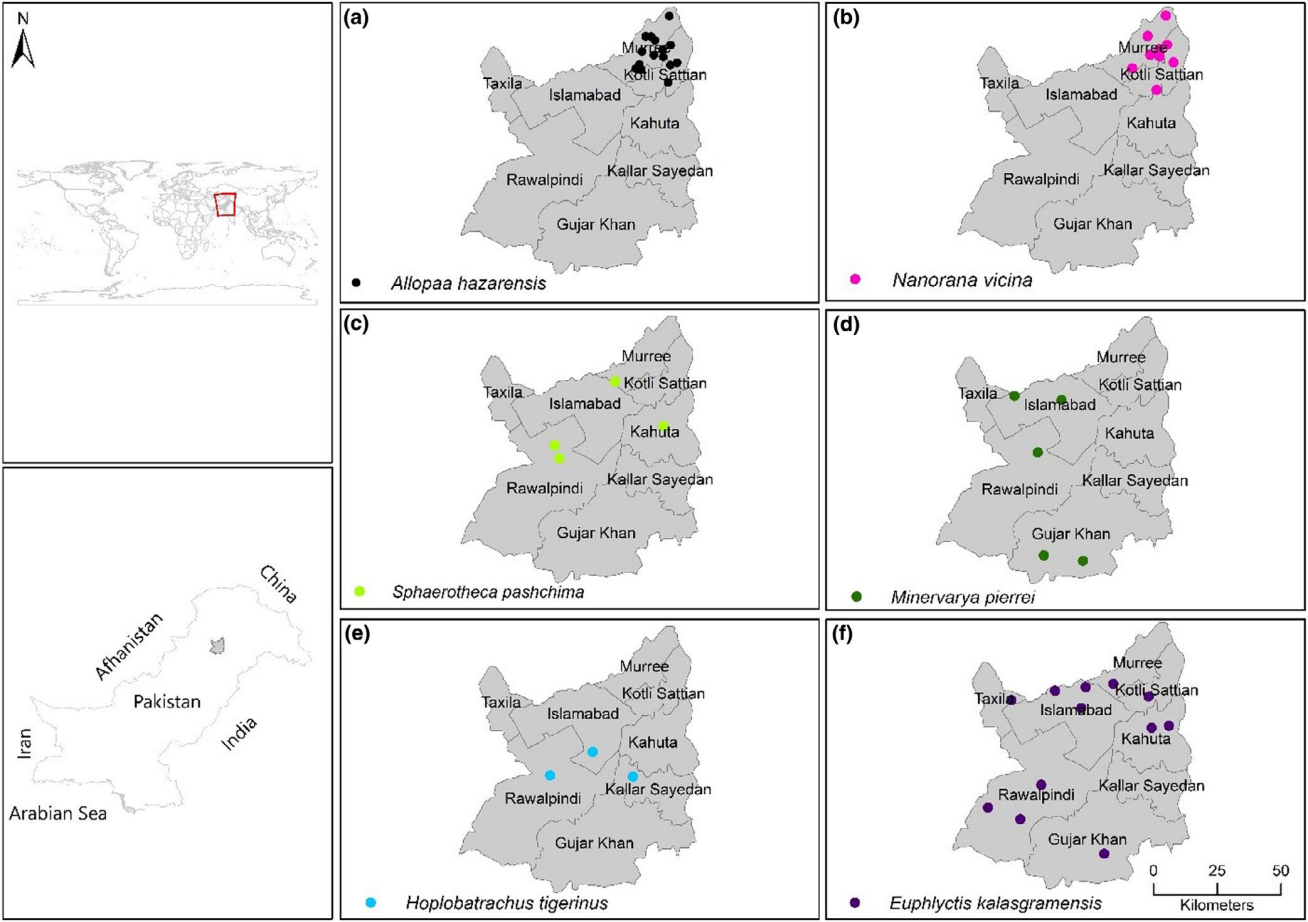
Map of the study area showing sampling locations of genus *Allopaa, Nanorana, Sphaerotheca, Minervarya, Hoplobatrachus* and *Euphlyctis* in Rawalpindi District, Punjab Province and Islamabad Capital Territory, Pakistan

The selected sampling area was Rawalpindi District (North Punjab) and Islamabad Capital Territory of Pakistan (Figures 1 and 2). The Rawalpindi District (33.4095°N, 72.9933°E) is located in the northwest of Punjab Province and covers an area of 5,286 km^2^. The area is rocky and has mostly scrub vegetation. Administratively, the district has seven tehsils (Rawalpindi, Gujar Khan, Kallar Syedan, Kahuta, Murree, Kotli Sattian, and Taxila). The areas experience a humid subtropical climate with long and hot summers, a short monsoon period, and mild wet winters (Chaudhry & Rasul, 2004). The average temperature ranges from 2°C in January to 38.6°C in the June. Tehsil Murree have an elevation of 804–2,291 m, with mean annual precipitation of 1,789 mm. The area features mainly subtropical Chir Pine (*Pinus roxburghii*) Forest (900–1,700 m elevation) and Himalayan Moist Temperate Forest. Other Tehsils such as Gujar Khan, Taxila, Rawalpindi, Kotli Sattian, and Kallar Syedan of Rawalpindi District have predominantly Subtropical Broad‐leaved Evergreen Forest or Scrub Forest with elevation <900 m (Sheikh & Hafeez, 2001). The district is drained by perennial and intermittent streams (Ahmed et al., 2020).

### 2.2 Field surveys

The specimens were collected from selected sites of study area (Figures 1 and 2) from February 2016 to October 2017. Field surveys were conducted in the morning (07:00–10:00) and evening (17:00–21:00). The length of field visits varied from a minimum of one day to a maximum of five days. All major habitats such as water, land, and vegetation were thoroughly searched, and only adult specimens were collected by hand or with dip net. The specimens were then brought to the Herpetology laboratory, Department to Wildlife Management, Pir Mehr Ali Shah‐Arid Agriculture University Rawalpindi, Pakistan.

### 2.3 Morphological identification

**FIGURE 3:**
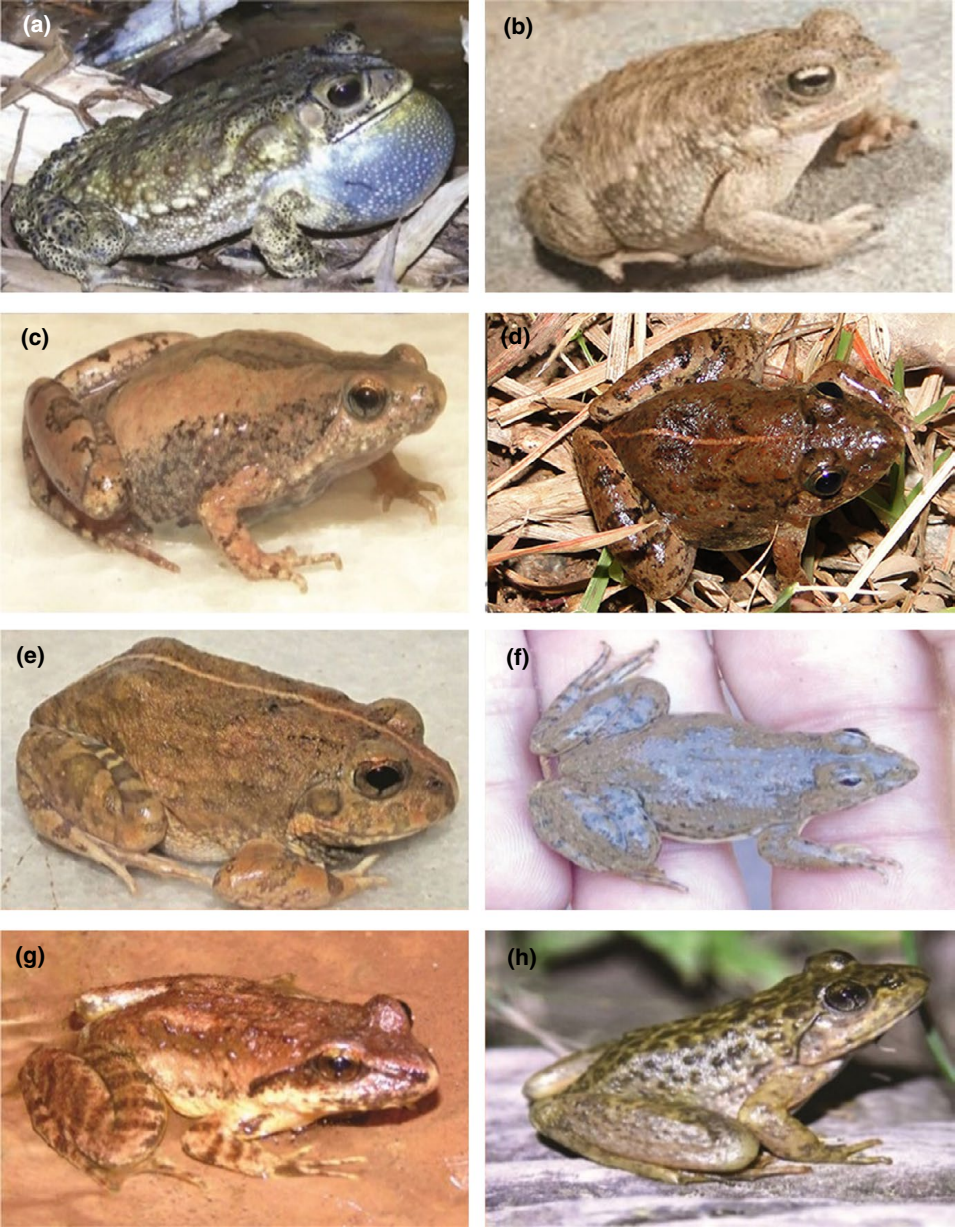
Anuran species of Rawalpindi District and areas of Islamabad. (a) South‐East Asian Toad (*Duttaphrynus melanostictus*); (b) Indus Valley Toad (*Duttaphrynus stomaticus*); (c) Nilphamari Narrow‐mouthed Frog (*Microhyla nilphamariensis*); (d) Pierre's Wart Frog (*Minervarya pierrei*); (e) Maskey's Burrowing Frog (*Sphaerotheca maskeyi*: synonym, *S* *. pashchima*); (f) Skittering Frog (*Euphlyctis kalasgramensis*); (g) Murree Hill Frog (*Nanorana vicina*); (h) Hazara Torrent Frog (*Allopaa hazarensis*). Photo credits: Muahmmad Rais and Muhammad Saeed

We collected nine anuran species, which included two toad species: *Duttaphrynus stomaticus* and *Duttaphrynus melanostictus,* and seven frog species: *Microhyla nilphamariensis*, *Allopaa hazarensis*, *Nanorana vicina*, *Sphaerotheca maskeyi* (synonym:*S. pashchima*)*, Minervarya pierrei, Hoplobatrachus tigerinus,* and *Euphlyctis kalasgramensis* (see Figure 3). The specimens were initially examined and identified based on morphological characters described in Khan (2006), Padhye et al. (2017), Howlader et al. (2015a, 2015b), and Howlader et al. (2016).

### 2.4 Molecular analysis

The anuran specimens were euthanized by using chloroform, and toe clips were removed and stored in 95% ethanol in sample tubes for genetic analysis. The voucher specimens were fixed and later preserved in 10% formalin solution. The preserved specimens were then deposited in the museum of Herpetology Laboratory, Department to Wildlife Management, PMAS‐AAUR. The general principles and guidelines of animal ethics were followed. The collected voucher specimens were not recognized as belonging to the threatened species and not listed in IUCN Red list or by CITES. Detailed lists of preserved voucher specimens are provided in Appendix 1.

#### 2.4.1 DNA extraction, amplification, and sequencing

We extracted DNA from stored tissue samples (toe clips) by using Promega Genomic DNA purification and extraction kit with the provided protocol for animal tissue. Quality of extracted DNA was assessed by Agarose Gel electrophoresis, and quantity of extracted DNA was calculated with Qubit 2.0 Fluorometer by using provided protocol of High Sensitivity Assay Kit. Two sets of primers (see Appendix 2) were used for the PCR amplification of 16S mitochondrial gene. For genus *Duttaphrynus, Microhyla, Sphaerotheca, Minervarya, Hoplobatrachus,* and *Euphlyctis,* we used the primer pair 16SAR, 16SBR (Palumbi, 1996), and for genus *Nanorana* and *Allopaa,* we used *the* primer pair 16SC, 16SD (Cannatella et al., 1998). The PCR protocol of previous publication (Palumbi, 1996) was followed with few modifications for this study. Two mitochondrial fragments were amplified in 25 µl volume reaction. The recipe for the master mix is 2 µl of genomic DNA template, 2.5 µl of 10× PCR buffer, 0.5 µl of dNTP, 0.125 µl of Taq DNA polymerase, 0.5µl of forward primer, 0.5µl of reverse primer, and 18.875 µl of distilled water. The annealing temperature of the primers was 50°C. The thermocycler settings were as follows: initial denaturation step with 4 min at 94°C, 40 cycles of denaturation 30 s at 94°C, annealing for 30 s at 50°C, and extension for 90 s at 72°C. Final extension at 72°C was conducted for 7 min. DNA amplification was confirmed by agarose gel electrophoresis. The resulting PCR products were then cleaned using the Promega DNA purification kit (Wizard^®^ SV Gel and PCR Clean‐Up System). The concentration of DNA was checked through NanoDrop Spectrophotometer (Invitrogen). The resulting PCR products were then sequenced in both directions using the same primers. Sanger sequencing was performed at the Institute for Cellular and Molecular Biology Core Facility, University of Texas Austin, Texas, USA. The sequences generated from the present study were deposited in the GenBank, and accession numbers are provided in Appendix 1.

#### 2.4.2 Data analysis

For reading, editing, and making consensus of forward and reverse sequences, the program Geneious (ver. R 7.1.9) (Kearse et al., 2012) was used. The new 16S sequences obtained were blasted on NCBI Nucleotide Blast Tool to identify and collect reference sequences of the same and closely related species. Sequences with more than 95% percentage similarity were retrieved and included in the analysis in order to find a good match. To analyze the taxonomic placements of our samples/species, we also included the representative samples of other species and genera of geographically linked species distributed throughout Pakistan, India, Bangladesh, Nepal, Sri Lanka, Iran, China, Japan, Taiwan, Uzbekistan, Greece, Turkey, Oman, Yemen, Indonesia, Malaysia, Vietnam, Thailand, Madagascar, and also from Himalayan range.

Alignments for each family, that is, Bufonidae, Microhylidae, and Dicroglossidae were prepared separately. A total of 37 samples including 8 newly sequenced and 29 from GenBank (two out‐groups *Ansonia longidigita* and *Ingerophrynus divergens*) were used for the phylogenetic analysis of Bufonidae. For the Microhylidae, 48 sequences were used including 4 newly sequenced samples and 44 sequences from GenBank (including two out‐groups *Uperodon systoma* and *Kaloula pulchra*). For Dicroglossidae, a total of 260 sequences including 62 newly sequenced samples and 198 from GenBank (including out‐groups *Rana asiatica* and *Rana catesbeiana*) were used in the analysis of Dicroglossidae. The details of sequences generated in the present study along with those recovered from GenBank are provided in Appendix 1.

Nucleotide sequences were aligned using MAFFT multiple sequence alignment program v. 7 with the option ‐‐auto that chooses the most appropriate algorithm for the data type (Katoh & Standley, 2013). The ambiguities, insertion, and deletion of single nucleotides from the sequences were manually edited using program Geneious (ver. R 7.1.9) (Kearse et al., 2012). Phylogenetic analysis of sequences was performed with maximum‐likelihood analysis (Bootstrap value 100) on the CIPRES Science Gateway V. 3.3 (Miller et al., 2010) using the software IQ‐TREE (Nguyen et al., 2015) with 1,000 ultrafast bootstraps approximation (Hoang et al., 2018). The model selection for each analysis tree was done as part of the run in IQ‐TREE (Kalyaanamoorthy et al., 2017) using the option ‐TESTONLY. The best‐fit model for Bufonidae family was TIM2+F+I+G4, for Dicroglossidae GTR+F+I+G4, and for Microhylidae TIM2e+I+G4. The consensus tree was calculated using SumTrees v.5.4.1 (Sukumaran & Holder, 2010). The tree with the highest maximum likelihood was selected, and the support from the bootstrap was mapped into that topology. The best value of maximum likelihood for Bufonidae was −3001.2908, for Dicroglossidae −9362.9580, and for Microhylidae −2110.5332.

To evaluate different strategy, an alignment that takes into account the secondary structure was made, with the option Q‐INS‐i of MAFFT online service: multiple sequence alignment, interactive sequence choice, and visualization (https://mafft.cbrc.jp/alignment/server/, Katoh et al., 2019). Based on secondary structure alignment, Bayesian phylogenetic inference (BI) (posterior probability 1) was performed in MrBayes ver. 3.2.6, (Ronquist et al., 2011). We ran the BI tree in CIPRES using MrBayes on XSEDE. We used the reversible jump Markov chain Monte Carlo approach in order to calculate a model of DNA substitution, which allows us to examine among 203 substitutions model using AIC (Akaike, 1974) and BIC (Schwarz, 1978). We ran the BI analysis using a set parameters of two runs with eight chains with length of 40 million generations, sampling a tree every 1,000 generations. The convergence and effective sample sizes (ESSs) >200 of the runs were seen in TRACER v.1.6.0 (Rambaut et al., 2014). A burn‐in was defined at 10%, by using SUMTREES v.4.2.0 (Sukumaran & Holder, 2010), and we discarded the burn‐in and calculated a maximum clade credibility tree (MCCT).

The pairwise genetic distances between species groups were estimated. Within the group mean distance, between‐group mean distance was calculated by using uncorrected *p*‐distances in the software MEGA. 7.0 (Kumar et al., 2016).

## 3 RESULTS

### 3.1 Phylogenetic analysis of Bufonidae (genus *Duttaphrynus*)

**FIGURE 4:**
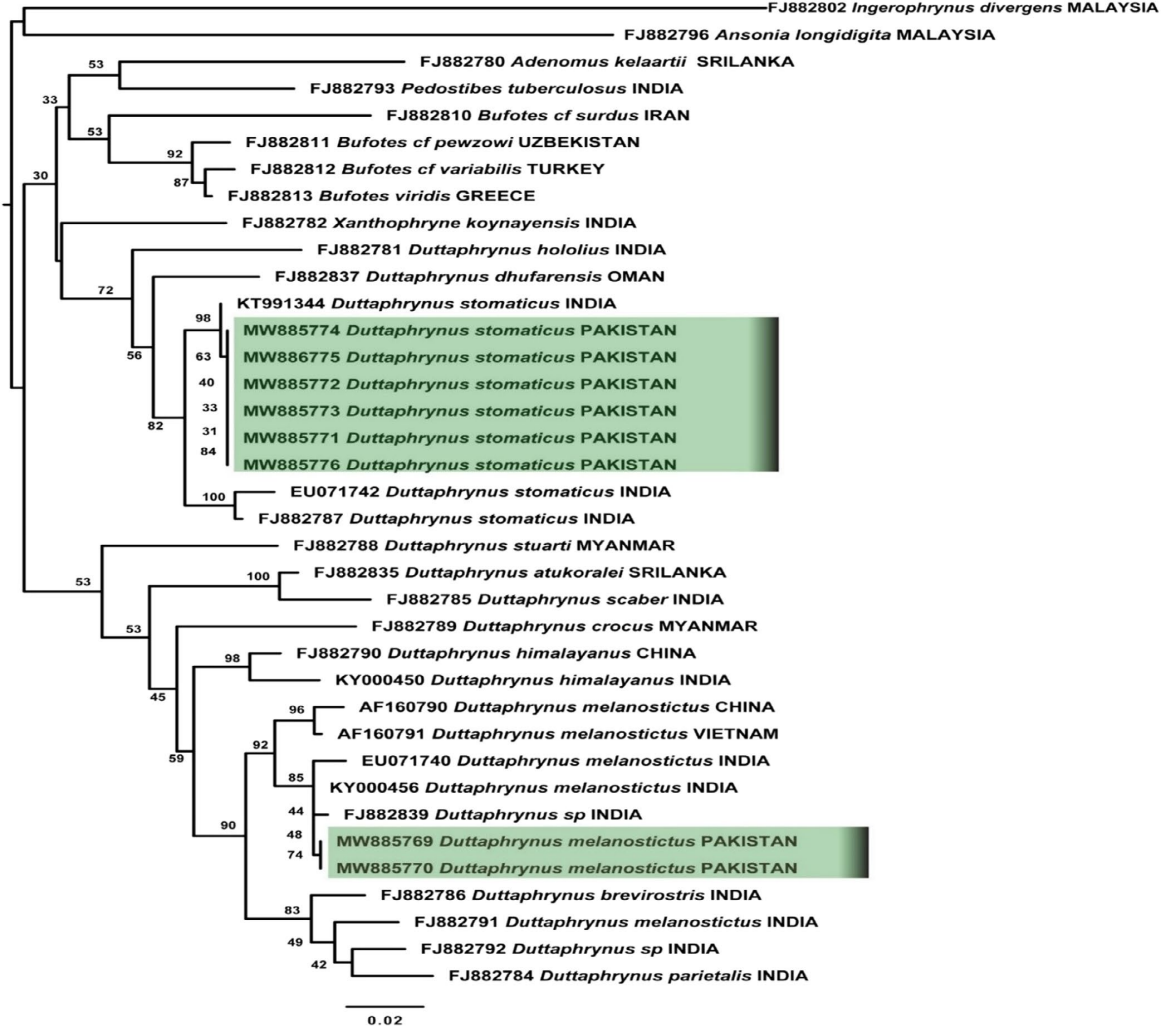
Maximum‐likelihood phylogeny from IQ‐TREE analyses based on the 16S rRNA, of genus *Duttaphrynus*, Family Bufonidae. The bootstrap percentages are indicated near each node. Sequences generated in the present study are highlighted. Details of samples are given in Appendix 1

We estimated phylogenies using the alternative alignments (primary and secondary structure), and found that the tree topologies are almost similar to each other. We conducted a maximum‐likelihood and Bayesian analyses for taxonomic identification of bufonid toads (Genus *Duttaphrynus*). The data matrix was comprised of 37 samples related to 20 species, including two out‐groups (*Ansonia longidigita* and *Ingerophrynus divergens*) and 18 in‐groups (*Adenomus kelaartii, Pedostibes tuberculosus, Xanthophryne koynayensis, Bufotes surdus, B. pewzowi, B. variabilis, B. viridis, Duttaphrynus melanostictus, D. stomaticus, D. brevirostris, D. atukoralei, D. dhufarensis, D. hololius, D. parietalis, D. scaber, D. stuarti, D. crocus, and D. himalayanus*) (Figure 4; Appendix 3). All the newly sequenced samples of *D. stomaticus* and *D. melanostictus* recovered in both ML and BI trees with bootstrap value for *D. stomaticus* clade were 82% and for *D. melanostictus* 92%. The posterior probability value for *D*. *stomaticus* was 0.96, and *D*. *melanostictus* was 0.98 (Figure 4; Appendix 3). As there were two subclades of *D*. *stomaticus* observed in the phylogenetic inference, newly generated samples shared a same subclade with genetically identical sample of *D*. *stomaticus* (India), as uncorrected p‐distance within the group is 0%. However, in the second subclade of *D*. *stomaticus* the uncorrected p‐distance within the group was 1.7%. Moreover, between these two subclades of *D*. *stomaticus* was 3.9%, which reflects some genetic variation within species (Figure 4; Appendix 3; Tables S1 and S2).

The newly generated sequences of *D. melanostictus* are more closer/identical to the Indian samples (with uncorrected p‐distance within group was 0.6%) as compared to *D. melanostictus* from China and Vietnam samples. The uncorrected p‐distance between the two clades (China, Vietnam) and (Pakistan, India) was 2.7% (Figure 4; Appendix 3; Tables S1 and S2). The results showed the genetic confirmation of newly generated sequences of *D*. *stomaticus* and *D*. *melanostictus* from Pakistan.

### 3.2 Phylogenetic analysis of Microhylidae (genus *Microhyla*)

**FIGURE 5:**
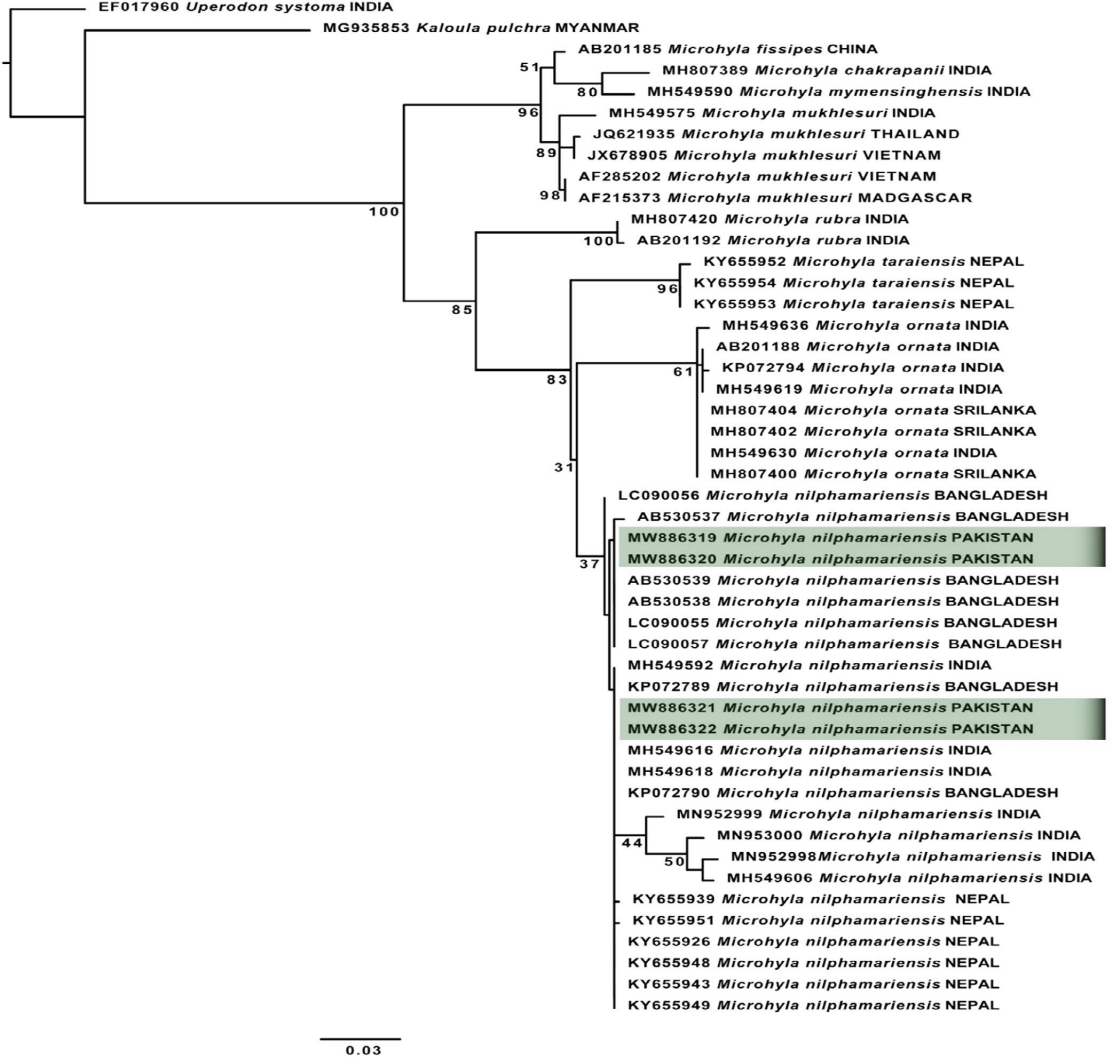
Maximum‐likelihood phylogeny from IQ‐TREE analyses based on the 16S rRNA, of genus *Microhyla*, Family Microhylidae. The bootstrap percentages are indicated near each node. Sequences generated in the present study are highlighted. Details of samples are given in Appendix 1

The maximum‐likelihood and Bayesian analyses of microhylid species were performed on data matrix comprised of 48 samples of 10 species including two out‐groups (*Uperodon systoma* and *Kaloula pulchra*) and 8 in‐groups of genus *Microhyla* (*M*. *ornata, M. chakrapanii, M. fissipes, M. mukhlesuri, M. mymensinghensis, M. nilphamariensis, M. rubra*, and *M. taraiensis*). The tree topologies were observed similar, based on both maximum‐likelihood and Bayesian analyses. The clade consisting of all sampled species of Genus *Microhyla* was highly supported in both ML and BI analyses (ML = 100 and PP = 1) (Figure 5; Appendix 4). However, the taxonomic status of our new samples of *Microhyla nilphamariensis* from Pakistan shared clade with the same species from India, Nepal, and Bangladesh which indicates its taxonomic status and wide distribution (BT = 37, PP = 0.83) (Figure 5; Appendix 4). The uncorrected p‐distance within the clade of *M. nilphamariensis* was 0.3%, which clearly indicates the genetic confirmation of these species (Table S4).

### 3.3 Phylogenetic analysis of family Dicroglossidae

**FIGURE 6:**
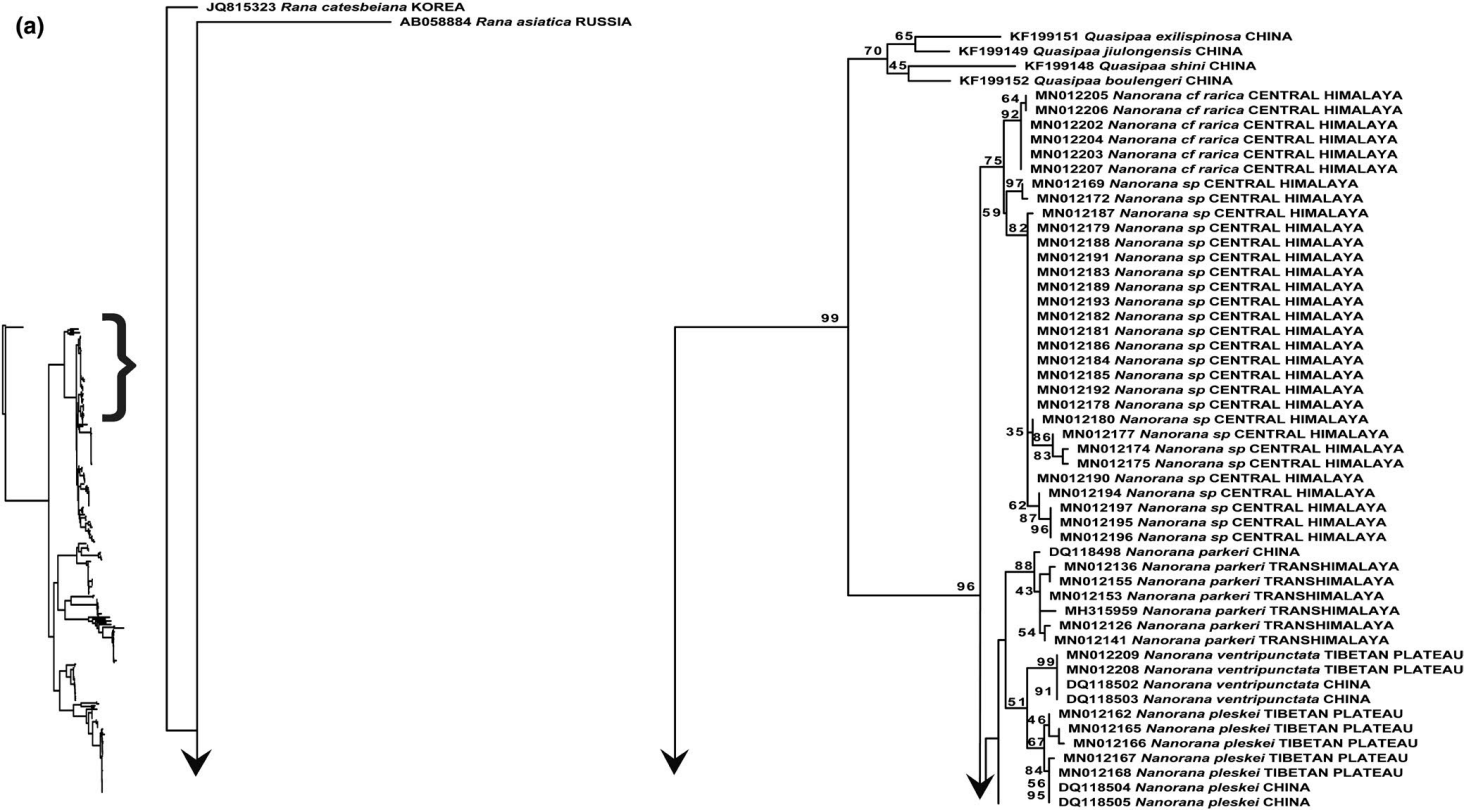
Maximum‐likelihood phylogeny from IQ‐TREE analyses based on the 16S rRNA, of genus *Nanorana*. The bootstrap percentages are indicated near each node. Details of samples are given in Appendix 1

**FIGURE 7:**
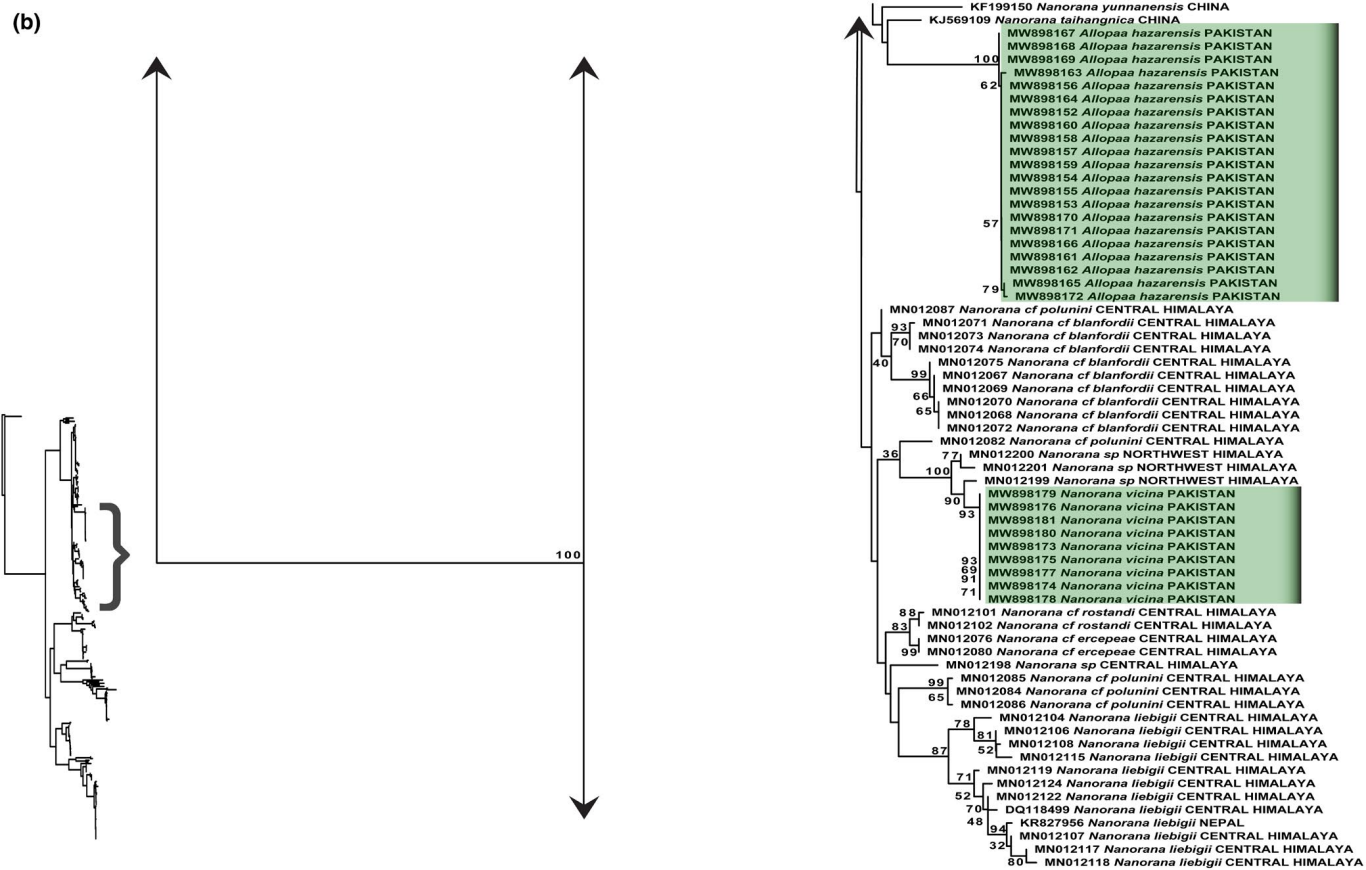
Maximum‐likelihood phylogeny from IQ‐TREE analyses based on the 16S rRNA, of genus *Nanorana* and *Allopaa*. The bootstrap percentages are indicated near each node. Sequences generated in the present study are highlighted. Details of samples are given in Appendix 1

The maximum‐likelihood and Bayesian analyses were conducted on final alignments of 260 sequences of 8 genera (*Allopaa, Nanorana, Quasipaa, Sphaerotheca, Fejervarya, Minervarya, Hoplobatrachus, and Euphlyctis*), whereas two species of genus *Rana* (*Rana catesbeiana* and *Rana asiatica*) served as out‐groups. We therefore inferred the phylogenetic analysis of our samples of *N. vicina* and *A*. *hazarensis* with 16S rRNA data of other species of genus *Nanorana* (*N*. *rarica, N. parkeri, N. ventripunctata, N. pleskei, N. yunnanensis, N. taihangnica, N. polunini, N. blanfordii, N. vicina, N. rostandi, N. ercepeae, N. liebigii,* and unidentified samples of genus *Nanorana* from the Himalayan range). The phylogenetic trees inferred from both maximum‐likelihood and Bayesian analyses were similar, and the tree topologies were well resolved for the *Quasipaa* and *Nanorana* species (including *Allopaa hazarensis* samples) with BT = 99 and PP = 1 (Figure 6; Appendix 5). The *N*. *vicina* and *A*. *hazarensis* samples were nested within the clade of genus *Nanorana* with nodal support of BT = 96 and PP = 1. The species taxonomic placement of *N*. *vicina* and *A*. *hazarensis* was highly supported by ML (BT = 100) and BI (PP = 1) analyses (see Figures 6 and 7; Appendices 5 and 6). Newly sequenced samples of *A*. *hazarensis* appeared as paraphyletic with respect to *Nanorana* species. *Allopaa hazarensis* samples were recovered as nested within genus *Nanorana,* which may lead to the possibility of having same genus (*Nanorana*) (Figures 6 and 7; Appendices 5 and 6). Furthermore, our samples of *N. vicina* were identical (uncorrected p‐distance of 0%) to the *N. vicina* from Northwest Himalayas (India: Himachal Pradesh), which depicts the existing distribution of *N. vicina* from Pakistan (type locality: Murree) to Himachal Pradesh (Figure 7; Tables S5 and S6). The uncorrected p‐distance between *N. vicina* and *A*. *hazarensis* was 2.1% (Table S5).

**FIGURE 8:**
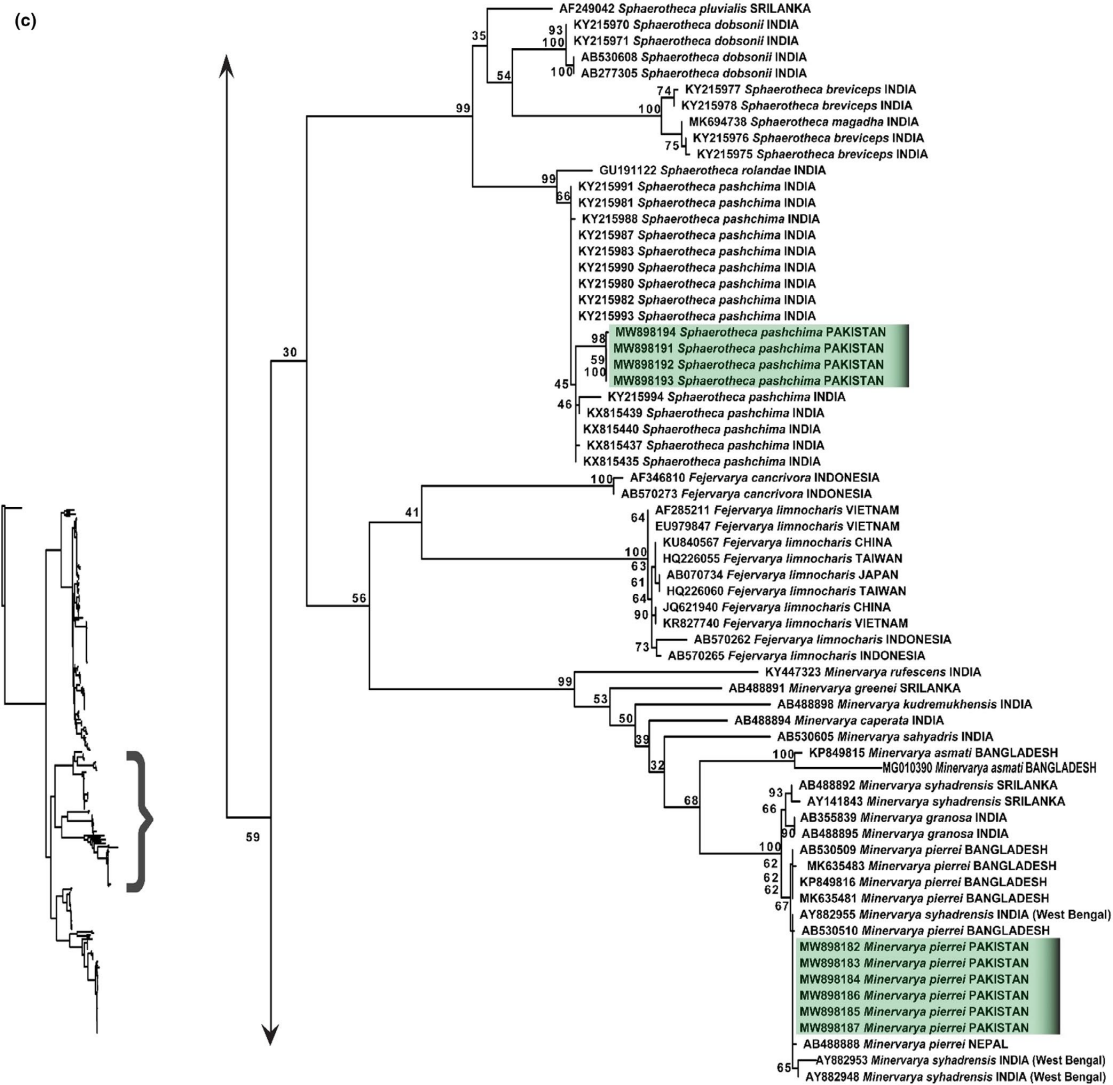
Maximum‐likelihood phylogeny from IQ‐TREE analyses based on the 16S rRNA, of genus *Sphaerotheca*, *Fejervarya,* and *Minervarya*. The bootstrap percentages are indicated near each node. Sequences generated in the present study are highlighted. Details of samples are given in Appendix 1

The maximum likelihood and Bayesian inference trees recovered all sampled species of genera *Sphaerotheca*, *Fejervarya,* and *Minervarya* in their respective clades (Figure 8; Appendix 7). All the sampled species of genus *Sphaerotheca* (*S*. *pluvialis, S*. *dobsonii*, *S*. *magadha*, *S*. *rolandae*, *S*. *breviceps,* and *S. pashchima*) appeared as an independent taxonomic species rank but with low branch support. New samples of genus *Sphaerotheca* from Pakistan appeared in the clade of *S*. *pashchima* (Figure 8; Appendix 7). The uncorrected p‐distance within group of *S*. *pashchima* was 0%; however, between *S. pashchima* and *S. breviceps,* it was 6.4% (Tables S5 and S6).

Species of genus *Fejervarya* (*F*. *cancrivora* and *F. limnocharis*) and *Minervarya* (*M*. *rufescens, M. greenei, M. kudremukhensis, M. sahyadris, M. caperata, M. asmati, M. granosa, M. syhadrensis,* and *M. pierrei*) appeared in their respective clade in both maximum likelihood and Bayesian inference (Figure 8; Appendix 7). The samples of *M*. *syhadrensis, M. granosa,* and *M. pierrei* were appeared in their respective subclades, under one main clade with maximum nodal support (BT = 100 and PP = 1). Newly generated sequences of *M*. *pierrei* are recovered as nested within *M*. *pierrei* clade. This species was previously misidentified in Pakistan as *F*. *limnocharis* (uncorrected p‐distance between *F. limnocharis* and *M. pierrei* was 7.9%). The uncorrected p‐distance between and within *M. pierrei* samples was observed as 0% (Tables S5 and S6). Therefore, we considered our samples as *M. pierrei* and reported first genetic record of this species from Pakistan.

**FIGURE 9:**
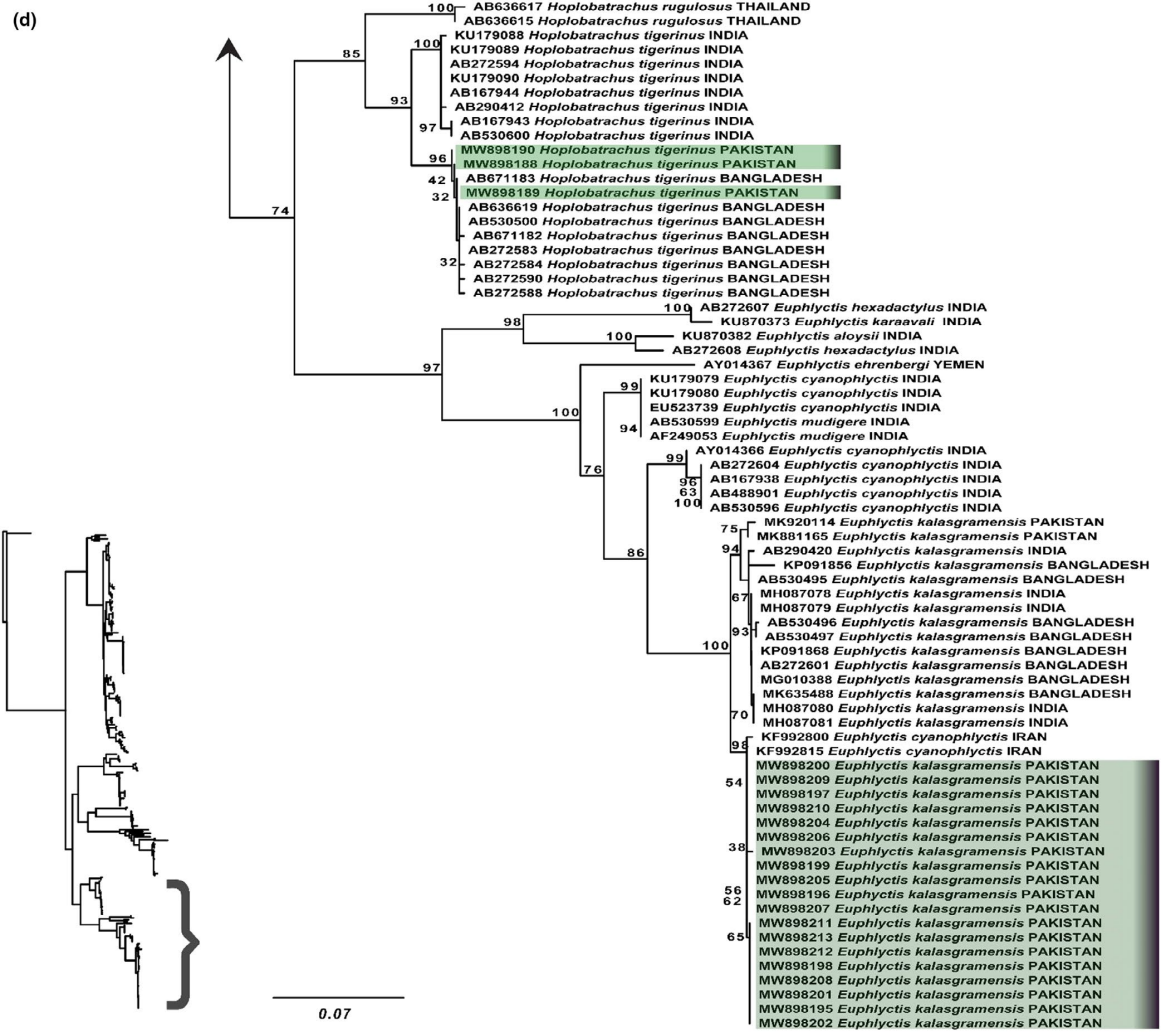
Maximum‐likelihood phylogeny from IQ‐TREE analyses based on the 16S rRNA, of genus *Hoplobatrachus* and *Euphlyctis*. The bootstrap percentages are indicated near each node. Sequences generated in the present study are highlighted. Details of samples are given in Appendix 1

For the genus *Hoplobatrachus*, we included species *H. rugulose* and *H. tigerinus* from its already established range of India and Bangladesh. Two haplotypes were observed in the *H. tigerinus:* One is from Indian population and other is Bangladeshi population. Our samples of *H*. *tigerinus* from Pakistan showed a well‐supported clade with Bangladesh samples of *H*. *tigerinus* (BT = 96 and PP = 1), which indicates validation of our samples as *H*. *tigerinus* (Figure 9; Appendix 8). The uncorrected p‐distance between *H*. *rugulose* and *H*. *tigerinus* was 6.3% and between haplotypes of Bangladesh and India was 1.6% (Table S5).

The highly supported clade of genus *Euphlyctis* (BT = 97 and PP = 1) included species of *E*. *hexadactylus, E. karaavali, E. aloysii, E. ehrenbergi, E. cyanophlyctis, E. mudigere,* and *E*. *kalasgramensis* (Figure 9: Appendix 8). Both maximum‐likelihood and Bayesian inference analyses of genus *Euphlyctis* strongly suggest that India, Pakistan, Bangladesh, and Iran populations of *E*. *cyanophlyctis* are split into four genetic lineages separated by nucleotide divergence (between group distance ranged from 0.9% to 3%) (Table S5). These lineages correspond to the clade from southern India, which include *E*. *mudigere,* the south Indian clade that we consider to be nominal *E*. *cyanophlyctis* because of its proximity to the type locality, the clade of *E*. *kalasgramensis* from specimens of Bangladesh, India (Assam), and Pakistan. The last clade was comprised of newly generated samples of Pakistan (Northern Punjab) and Iran (Figure 9; Appendix 8). Our samples of *Euphlyctis* appeared as a sister clade (BT=98; PP=1) of *E. kalasgramensis* clade constituting samples from Pakistan, India, and Bangladesh (Figure 9; Appendix 8). However, the uncorrected p‐distance between two sister clades of *E*. *kalasgramensis* was 2.1% (Table S5). Furthermore, two samples of Iran shared the same clade with our samples with 0% genetic divergence (Table S6).

## 4 DISCUSSION

We conducted the first comprehensive molecular study on anurans of Pakistan, which entailed about 37% of the known anuran species of the country, including eight genera and nine species. The maximum‐likelihood analysis based on standard alignment and Bayesian analysis based on secondary structure alignment validated the taxonomic status of *D*. *stomaticus, D. melanostictus*, *M*. *nilphamariensis, S. maskeyi* (synonym*: S. pashchima*), *H. tigerinus, E. kalasgramensis,* and *A*. *hazarensis* in Pakistan. We provided the genetic record of *N*. *vicina* (endemic to South Asia), for the first time from Pakistan and confirmed their species taxonomic ranks. We also reported first genetic record of genus *Minervarya* (*M. pierrei*) from Pakistan. This species were previously identified as *F. limnocharis* from the Rawalpindi District and Islamabad Capital Territory, Pakistan.

There are 12 true toad species of family Bufonidae, reported from Pakistan. Pakistani toad species can be represented in three groups *viridis, stomaticus,* and *melanostictus* (Khan, 1976). *Duttaphrunus stomaticus* and *D*. *melanostictus* toads can be morphologically identified by their parotid glands, rough skin with warts, and unwebbed toes. However, they can be distinguished from each other by distinct cranial crest present on head of *D*. *melanostictus* (Khan, 2006). *Duttaphrynus stomaticus* (Lütken, 1864) and *D. melanostictus* (Schneider, 1799) both include in a taxonomic framework of a monophyletic group of *Duttaphrynus* (Dubois & Ohler, 1999). Regardless of several studies on phylogenetic relationships in the family Bufonidae elsewhere, species from Indian subcontinent, especially from Pakistan, have not been studied in detail (Van Bocxlaer et al., 2009). In addition to many species with uncertain taxonomic affiliations, *D. stomaticus* and *D. melanostictus* (previously labeled as *Bufo*) require more detailed phylogenetic evidence (Van Bocxlaer et al., 2010). Van Bocxlaer et al. (2010) reported *D*. *stomaticus* being limited to the Western Ghats which is not true (Akram et al., 2015; Portik & Papenfuss, 2015). The previous sampling was limited to India leading to a sampling gap across Pakistan, a region that contains many widespread species in the genus *Duttaphrynus* and *Bufotes* (Portik & Papenfuss, 2015). Hussain et al. (2020) recently confirmed the taxonomic status of *D. stomaticus* and *D*. *melanostictus* from Punjab, but using one sample of each species, with limited dataset. We hereby presented an extensive dataset by incorporating 16S molecular data of 20 species, to confirm the taxonomic position of *D*. *stomaticus* and *D. melanostictus* with respect to other species of same genus. Our phylogenetic inferences yielded genetic confirmation of these two toads from Pakistan. The present study is largely in agreement with Portik and Papenfuss (2015) who recorded the species from Tharparkar (Sindh) and Lasbela (Baluchistan), and reported the possible distribution of *D*. *stomaticus* throughout Pakistan. We, however in this study provided genetic samples from northern Punjab, Pakistan and confirmed taxonomic status of *D*. *stomaticus*.

The species complex of *D*. *melanostictus* also entails taxonomic revision. It exhibits a wide geographical range (Wogan et al., 2016). The ancestral range for *D*. *melanostictus* is estimated to be the Myanmar–China border (Wogan et al., 2016). We validated the taxonomic status of *D. melanostictus* based on our maximum‐likelihood and Bayesian analyses, which inferred that newly reported samples from the study area are closely related to the Indian samples with uncorrected p‐distance within group of 0.6% (Table S2). The genetic divergence of 1.7% was observed between the two clades: (China, Vietnam) and (Pakistan, India) (Table S1). This genetic variation within species was previously reported by Khan (2001), who differentiated this species of *D*. *melanostictus* from South‐East Asian congeners based on morphological parameters and ranked Pakistani samples as a subspecies named *D. melanostictus hazarensis*. Our genetic data are also in agreement with this existing variation within this species across its range. Another study by Mulcahy et al. (2018) also indicated this genetic variation but referred all the samples as *D*. *melanostictus* until this species complex is revised.

Microhylid species are believed to be one of the most challenging taxonomical group of microhylid frogs due to their small size, conserved morphology, and widespread distribution of its members across Asia (Garg et al., 2018); Matsui et al., 2005, 2011). Molecular data have doubled the number of recognized *Microhyla* species found in South Asia (Hasan et al., 2014; Howlader et al., 2015a; Khatiwada et al., 2017; Seshadri et al., 2016; Vineeth et al., 2018; Wijayathilaka et al., 2016) and delineation of already known taxa (Garg et al., 2018; Hasan et al., 2012; Matsui, 2011; Matsui et al., 2005, 2011; Yuan et al., 2016), therefore elevating the importance of this region for *Microhyla* diversity.

*Microhyla ornata* was assumed to be broadly distributed species throughout India, Bangladesh, Bhutan, Nepal, Pakistan, and Sri Lanka (Dutta et al., 2008), on the basis of century old range assumptions (Boulenger, 1882; Parker, 1934) and previous literature (Matsui et al., 2005, 2011). The checklists and records lacked vouchers or molecular information (Dinesh et al., 2009; Khan, 2006; Mathew & Sen, 2010). The genetic variations within populations of Microhylid species were first examined by Matsui et al. (2005), among three geographical regions of South Asia, South‐East Asia, and East Asia. The study allocated and restricted the name *M*. *ornata* to the South Asian populations. Matsui et al. (2011) provided insights on phylogenetic relationships among *Microhyla* frogs across their known range; however, the South Asian members especially from Pakistan were not included. The South Asian *M*. *ornata* was recently described as a species complex (Hasan et al., 2012), followed by the description of four new and closely related species *M*. *mymensinghensis* (Hasan et al., 2014), *M*. *mukhlesuri* (Hasan et al., 2014), *M*. *nilphamariensis* (Howlader et al., 2015a), and *M*. *taraiensis* (Khatiwada et al., 2017). The recent reports of three new Microhylid species from India (*M*. *mukhlesuri, M. mymensinghensis,* and *M*. *nilphamariensis*) (Garg et al., 2018) lead to the urgency of the detailed sampling and genetic confirmation from its entire range. Based on extensive sampling by Wijayathilaka et al. (2016), *M*. *ornata* actually exhibits a narrow distribution limited to Peninsular India, more specifically in the states of Kerala, Karnataka, Tamil Nadu, Maharashtra, and Andhra Pradesh. Despite being a relatively newly identified microhylid, *M*. *nilphamariensis* is now genetically confirmed from the Western Ghats, Eastern Ghats up to Central India, East India, North India, Northeast India, Nepal, and Bangladesh (Garg et al., 2018).

The genus was represented in Pakistan by *M*. *ornata* (Duméril & Bibron, 1841); however, in view of the overall distribution and diversity of the genus based on genetic data (Garg et al., 2019; Gorin et al., 2020), populations from Pakistan are *M. nilphamariensis*. It was misidentified by previous studies such as Khan (2006), as previous research was only based on morphological examination. Our phylogenetic analysis inferred a resolved topology by showing an independent species taxonomic rank of each Microhylid species (Figure 5; Appendix 4). We confirmed existence of *M*. *nilphamariensis* in the study area, as our samples formed the same clade with *M. nilphamariensis* samples from India and Bangladesh. This evidence was supported by less support in ML analysis (<50%) but with high support in BI (0.83) (Figure 5; Appendix 4). As, *M*. *nilphamariensis* was misidentified as *M. ornata* from Pakistan, high genetic divergence (uncorrected p‐distance 5%) was also observed between *M. nilphamariensis* and *M*. *ornata,* which leads to recognition of this species as *M. nilphamariensis* (Table S3). Our results are in agreement with Howlader et al. (2015a), who reported genetic divergence of *M*. *nilphamariensis* with its congeners (5.7% to 13.2%). In a recent study, Jablonski et al. (2020) also revealed the species status of the genus from Pakistan (Islamabad and Northern Punjab) and reported the populations from the country as *M*. *nilphamariensis*. Based on recent studies of Garg et al. (2019) and Gorin et al. (2020), Jablonski et al. (2020) also reported the absence of *M*. *ornata* from Pakistan. *Microhyla nilphamariensis* species can also be morphologically characterized based on its dark brown diamond‐shaped marking on dorsal side, dark streak from back of eyes to shoulders, lateral bands from tip of snout to the groin on either side of the body, blackish‐brown mottling on throat, chest and margins of the belly, and indistinct inner metacarpal and metatarsal tubercles (Howlader et al., 2015a). We therefore in accordance with Jablonski et al. (2020) reported a significant range extension westward by confirmation of its presence in Pakistan that prior to this was only reported from northern Bangladesh, central and eastern Nepal, northwestern Uttar Pradesh, possibly northern Rajasthan, Kashmir, Assam, Western Ghats region of Maharashtra, Karnataka, and Kerala (Garg et al., 2018, 2019; Howlader et al., 2015a; Khatiwada et al., 2017).

We provided the first genetic records of *N*. *vicina* from Pakistan by sampling from its type locality (Murree). *Nanorana vicina* (Stolickza, 1872) is a least studied anuran species from Pakistan. After its initial reports from Pakistan by Dubois (1976) from Azad Kashmir, Baig (2002) from Ayubia, Masroor (2011) from Margalla Hills National Park, and Rais et al. (2014) from Murree (type locality), the genetic validity of this species was still lacking. Previously this species was reported based on its morphological characters. The morphological diagnostic features included brownish smooth body dorsum with a few tubercles on flanks, dark bars on forearm, thighs and shank, distinct sooty stripes from snout to angle of jaws (see Figure 3; Khan, 2006; Rais et al., 2014). Hofmann et al. (2019) performed phylogeny of genus *Nanorana* from vast Himalayan range, but there was a sampling gap from Pakistan. They did not confirm the taxonomic status of several samples of the Himalayan range and left them as unidentified species. Latterly, Hofmann et al. (2021) referred *Nanorana* sp. samples from Himachal Pradesh as *N*. *vicina,* based on morphological (photographic) identifications. However, in the present study, we validated the taxonomic status of *N*. *vicina* genetically, as our samples of *N*. *vicina* appeared identical (uncorrected p‐distance of 0%) to the samples of *Nanorana* (MN012201, MN012200, and MN012199) collected from Northwest Himalayas (India: Himachal Pradesh) in the study of Hofmann et al. (2019). Moreover, our maximum‐likelihood and Bayesian analyses showed the nesting of all species of genus *Nanorana* under one main clade with branch support of 96 (BT) and 1 (PP) (Figures 6 and 7; Appendices 5 and 6). So, we in accordance with Hofmann et al. (2021) referred these samples as *N*. *vicina*. The existing distribution of this South Asian endemic species from Pakistan (Type locality: Murree) to India: (India: Himachal Pradesh) northwest Himalayas is now evident based on our phylogenetic analysis (Figures 6 and 7; Appendices 5 and 6). The geographical distribution of *N*. *vicina* in the west Himalayan range is also supported by several studies such as Sclater (1892), who reported its distribution range to Shimla, India. Litvinchuk et al. (2017) also reported its distribution in the west Himalayan range (Himachal Pradesh). As this species was understudied and had no previous genetic information, this study would be used as a reference for the future validation of conspecifics throughout its range.

*Allopaa hazarensis* (Dubois & Khan, 1979), which is endemic to Pakistan, was first placed in the supergroup of *Paa liebigii* (Dubois & Khan, 1979). Latterly, based on its unique combination of morphological characters, Ohler and Dubois (2006) proposed a separate genus for this species. In a recent study, Hofmann et al. (2021) conducted the first phylogenetic analysis based on genetic sampling of *A*. *hazarensis* from foothills of Kashmir Himalaya, which also includes its type locality (Dutta, District Mansehra). In this study, we included samples from same geographical range, that is, Murree (North Punjab), and our results showed genetic resemblance of *A. hazarensis* with all congeners of *Nanorana* and recovered as paraphyletic with respect to all sampled *Nanorana* species (Figures 6 and 7; Appendices 5 and 6), with genetic distance of 2.1% between *N*. *vicina* and *A*. *hazarensis* (Table S5). Hofmann et al. (2021) also reported this paraphyletic relationship of *Allopaa* with respect to genus *Nanorana*.

The separate genus of *Allopaa* which was described by Ohler and Dubois (2006) on morphological basis has appeared as nested within genus *Nanorana* by genetic analysis. *Allopaa hazarensis* also share its morphological characters with *N*. *vicina,* except having grayish dorsum with a superimposed network of dark olive green color, with horny spinules on dorsal and lateral sides and well‐developed male secondary sex characters (nuptial spines) (Dubois & Khan, 1979) (see Figure 3). Both *A*. *hazarensis* and *N*. *vicina* share their habitat (freshwater streams) at higher elevation (>1,000 m) (Ahmed et al., 2020). So, based on our phylogenetic inferences, morphological characters, and habitat preferences, we doubt on the validity of generic status of *A*. *hazarensis*. As our data were not enough to resolve this taxonomic issue, we suggest sequencing of additional mitochondrial and nuclear genes in the future studies to get a better resolution. As a least studied genus, which is exclusive to Pakistan, with no other documented species till date, the genus is particularly important for Pakistan. However, to prevent taxonomic instability, we are hesitant to propose any taxonomic changes until further evidence is available.

Our results based on maximum‐likelihood and Bayesian analyses agreed with previous studies by recovering three main subclades, corresponding to (a) genus *Sphaerotheca,* (b) South Asian clade (*Minervarya*), and (c) South‐East Asian clade (*Fejervarya*) (Dinesh et al., 2015; Hasan et al., 2014; Howlader, 2011; Kotaki et al., 2008; Kuramoto et al., 2007; Pyron & Wiens, 2011; Sanchez et al., 2018). All the sampled species in our dataset of genus *Sphaerotheca* (*S*. *pluvialis, S*. *dobsonii*, *S*. *magadha*, *S*. *rolandae*, *S*. *breviceps, S. pashchima*) recovered to have an independent taxonomic species rank albeit with low branch support (Figure 8; Appendix 7). *Sphaerotheca breviceps,* which is endemic to South Asia, was considered as a species complex (Dubois, 1983; Dutta, 1986), but Padhye et al. (2017) restricted its distribution range to the eastern coastal plains of India and described a new species, *S*. *pashchima* from western and northern India, which was previously misidentified as *S*. *breviceps*. *Sphaerotheca pashchima* is considered as a morphologically and genetically distinct species from western Maharashtra, Gujarat, and Karnataka, after its comparison with topotypic material of *S*. *breviceps*. *Sphaerotheca pashchima* differs from *S*. *breviceps* by minor differences such as rounded snout, second finger length equal to or less than fourth finger length; first finger length less than third finger (Padhye et al., 2017). Khatiwada et al. (2021) declared *S*. *pashchima* as a synonym of *S. maskeyi* (Schleich and Anders, 1998). Jablonski et al. (2021) also designated this species as *S*. *maskeyi,* from Himalayan foothills of Pakistan, based on two genetic samples collected from Khyber Pakhtunkhwa Province, Pakistan. In this study, *Sphaerotheca* sp. collected from north Punjab were clustered within *S*. *pashchima* clade, with uncorrected p‐distance of 0%. However, genetic distance between *S*. *pashchima* and *S. breviceps* was 6.4% (Tables S5 and S6). Our results are in congruent with successive studies of Padhye et al. (2017), Jablonski et al. (2021), and Khatiwada et al. (2021), and we believe the presence of *S*. *maskeyi* (synonym:*S*. *pashchima*) in North Punjab and these molecular studies resolved the taxonomic status of this species complex. However, by extensive genetic sampling in future studies, we expect more species of *Sphaerotheca* to be discovered from the region.

The frogs from genus *Fejervarya* are morphologically similar to many new morphologically cryptic (but genetically distinct) species (Sanchez et al., 2018). Recent taxonomic rearrangement of *Fejervarya* treats South Asian and South‐East Asian taxa as separate genera. The South‐East Asian species *Rana limnocharis* Gravenhorst 1829 (the type species of *Fejervarya*) is the first‐described genus named in the group (Frost, 2019). *Fejervarya Limnocharis* was reported by earlier workers (such as by Akram et al., 2015; Khan, 2006; Pratihar et al., 2014; Rais et al., 2012) from Pakistan, merely on basis of morphological characters. In the present study, we did first ever phylogenetic analysis based on genetic sampling from Pakistan. *Minervarya* species from the North Punjab which was misidentified previously as *F. limnocharis* is actually *M*. *pierrei* with 7.9% uncorrected p‐distance between groups (Table S5). The second described clade (primarily South Asian taxa) contains the type species of both *Minervarya* and *Zakerana* (*Minervarya sahyadris*) Dubois et al. (2001), and *Rana limnocharis syhadrensis* Annandale (1919), from which *Minervarya* was described earlier and have taxonomic priority on *Zakerana*. *Minervarya* placement within the South Asian clade is also confirmed by our both maximum‐likelihood and bayesian analyses, as our newly sequenced samples were nested within *Minervarya* genus with maximum branch support. The samples of *M*. *syhadrensis, M. granosa,* and *M. pierrei* were appeared in their respective subclades, under one main clade with maximum nodal support (BT = 100 and PP = 1) (Figure 8; Appendix 7). Our results are in congruent with Köhler et al. (2019), who also grouped these species in one main clade by using Automated barcode recovery method.

Our sequences were placed in *M*. *pierrei* group, and the taxonomic status of our samples was also validated by having 0% uncorrected p‐distance within *M*. *pierrei* samples (Tables S5 and S6). Phuge et al. (2020) examined two *M*. *syhadrensis*‐like species, which they named as types A and B and comparison of these types with the type specimen of *M*. *syhadrensis* from Pune district (India) through morphological, call pattern, and phylogenetic analysis referred type A as *M*. *syhadrensis,* whereas the other type was referred as *M*. *pierrei*/ *M*. *Agricola* complex. We also analyzed these samples having accession numbers AY882955, AY882953, and AY882948 originated from India (West Bengal). Therefore, we in accordance with Phuge et al. (2020) referred samples originated from Pakistan, Bangladesh, Nepal, and west Bengal (India) as *M*. *pierrei* clade (Figure 8; Appendix 7). This species can also be identified through morphological features of pointed snout, dorsum postulate, and vocal sacs, and markings on throat are laterally dark and medially pale, mid‐dorsal line with constant width from snout to vent (see Figure 3) (Howlader et al., 2016). Furthermore, as type locality of *M*. *pierrei* is east Nepal, which is in closer proximity with Northern Punjab (Pakistan). Therefore, by considering geographical proximity, previous study of Phuge et al. (2020), our phylogenetic inferences (Figure 8; Appendix 7) and morphological characters, we identified our samples as *M*. *pierrei*. We believe that more extensive phylogenetic datasets are required in order to provide genetic evidences of *M. syhadrensis* and *M. granosa* in Pakistan.

The phylogenetic analysis of genus *Hoplobatrachus* showed that the *Hoplobatrachus* samples from Pakistan (North Punjab) were similar to Bangladeshi samples of *H*. *tigerinus* with 0% genetic divergence; however, 1.6% genetic divergences were observed between Indian and Bangladeshi clades (Tables S5 and S6) (Figure 9; Appendix 8). Similarly, in a recent study of Khatiwada et al. (2021), Nepalese samples were appeared as closely related to Bangladesh samples as compared to Indian subclade, which indicates that the *H*. *tigerinus* present in geographical range of Pakistan (North Punjab), Bangladesh, and Nepal are genetically identical; however, there is some genetic variation exist between these and Indian lineage.

The genus *Euphlyctis* (Schneider, 1799) is one of the most widespread in southern Asia. It comprises of seven extant species: Description of new species has been reported in this genus from past few years, that is, *E. mudigere* (Joshy et al., 2009) from Southern India and Sri Lanka, *E*. *kalasgramensis* (Howlader et al., 2015b) from the Barisal district of Bangladesh, and *E*. *karaavali* (Priti et al., 2016) from West Coastal Plains of India. *E*. *cyanophlyctis*, the most common species, exhibits high degree of morphological similarity with other species of the genus (Joshy et al., 2009). Its distribution is known from Southeastern Iran, Southern Afghanistan, Pakistan, Nepal, Bhutan, India, Sri Lanka, Myanmar, Malaysia and Vietnam. Phylogenetic analyses of genus *Euphlyctis* strongly suggest that India, Pakistan, Bangladesh, and Iran populations of *E. cyanophlyctis* are split into four genetic lineages separated by nucleotide divergence (between groups uncorrected p‐distance ranged from 0.9% to 3%) (Table S5). These lineages correspond to the separate clades, clade 1 corresponds to southern India, which include *E. mudigere*, and second clade is the south Indian clade that we consider to be nominal *E*. *cyanophlyctis* because of its proximity to the type locality. Third clade constitutes samples of *E. kalasgramensis* from Bangladesh, India (Assam), and Pakistan and the last clade with newly generated samples of Pakistan (Northern Punjab) and Iran (Figure 9; Appendix 8). Two samples (KF992800 and KF992815) from Iran in study of Khajeh et al. (2014) are genetically identical to our samples (0% uncorrected p‐distance with our samples) (Table S6). Our results are in agreement with the study of Khajeh et al. (2014), which suggested that samples from Iran population of *E. cyanophlyctis* are more closely related to Bangladeshi population, as compared to south Indian population. This argument became strengthened when Howlader et al. (2015b), described a new species *E. kalasgramensis* from Bangladeshi population.

The existence of *E. kalasgramensis* from Pakistan was also reported by Ali et al. (2020) from Kasur (Punjab), Pakistan, based on molecular and morphological evidences, but with limited dataset. The identification features of *E*. *kalasgramensis* include the following: the body dorsum without longitudinal folds, plain whitish ventral coloration and limbs with incomplete dark bands (Howlader et al. (2015b) see Figure 3. In the present study, we analyzed extensive datasets by adding other congeneric species of this genus in our phylogenetic inferences. Our samples of *Euphlyctis* appeared as a sister clade of *E*. *kalasgramensis* comprised of samples originated from Pakistan, India, and Bangladesh. The genetic divergence of 2.1% (uncorrected p*‐*distance) was observed between these two sister clades (Table S5). However, we suggest detailed taxonomic study exclusively for this genus by including more mitochondrial and nuclear genes to clarify the presence of possible cryptic species under the taxonomic rank of *Euphlyctis*.

## 5 CONCLUSIONS

This study was aimed ‐ to document the species taxonomic status based on 16S rRNA. The phylogenetic analysis of South Asian anuran species was performed, in which new geneticsamples obtained from Pakistan and their respective congeners retrieved from GenBank were included. Our results based on maximum‐likelihood and Bayesian analyses of 16S rRNA data validated the taxonomic status of nine anuran species which belong to eight genera from Pakistan. These species include *Duttaphrynus stomaticus, Duttaphrynus melanostictus*, *Microhyla nilphamariensis*, *Allopaa hazarensis*, *Nanorana vicina, Sphaerotheca maskeyi*(synonym: *S. pashchima*)*, Minervarya pierrei, Hoplobatrachus tigerinus,* and *Euphlyctis kalasgramensis*. We reported the first genetic record of genus *Minervarya* (*M*. *pierrei*), This species was misidentified previously in Pakistan as *Fejervarya limnocharis*. Furthermore, we provided the first genetic records of *N. vicina* from its type locality (Murree), which will provide a baseline data for this understudied species.

### 5.1 Recommendations

Systematic surveys have never been conducted in Pakistan to document amphibian diversity of the country. A deep review is still necessary to resolve the taxonomy morphologically undistinguishable putative species complexes in the future studies.

The known fauna was based on work carried by individuals within their own capacity without any institutional setup and coordination among the researchers. Either individuals working in academic and research institutions gather samples or their peers bring them the samples for species identification. Utilizing their full capacity and understanding, they try to identify the species. Amphibians in Pakistan have failed to find any place in policy and legislation. We suggest carrying out extensive surveys throughout the country for the collection of specimens and their genetic analysis in future studies. These analyses will confidently resolve the taxonomic issues caused due to morphologically undistinguishable species. This would result in proper scientific documentation of amphibians of Pakistan. Many new species, some of them might be exclusive to Pakistan, are expected to be discovered, and taxonomic status of other species would be resolved.

## CONFLICT OF INTEREST

The authors declare no conflict of Interest. The funding agencies had no role in design of study, in the collection, analysis, interpretation of data, in the writing of the manuscript, or in the decision to publish the results.

## AUTHOR CONTRIBUTIONS

**Ayesha Akram:** Conceptualization (equal); Data curation (lead); Formal analysis (lead); Funding acquisition (equal); Investigation (lead); Methodology (lead); Project administration (equal); Resources (lead); Software (lead); Supervision (equal); Validation (lead); Visualization (lead); Writing‐original draft (lead); Writing‐review & editing (lead). **Muhammad Rais:** Conceptualization (equal); Funding acquisition (lead); Project administration (equal); Resources (equal). **Karem Lopez‐Hervas:** Data curation (supporting); Formal analysis (equal); Methodology (equal); Software (equal); Supervision (supporting). **Rebecca D. Tarvin:** Conceptualization (supporting); Data curation (equal); Formal analysis (supporting); Methodology (supporting); Resources (supporting); Software (supporting); Writing‐review & editing (supporting). **Muhammad Saeed:** Data curation (supporting); Project administration (supporting). **Daniel I. Bolnick:** Conceptualization (lead); Data curation (lead); Project administration (lead); Resources (lead); Supervision (lead); Writing‐review & editing (equal). **David C. Cannatella:** Conceptualization (lead); Data curation (lead); Formal analysis (lead); Methodology (equal); Resources (supporting); Software (supporting); Supervision (lead); Validation (lead); Writing‐review & editing (equal).

## Supporting information

Tables S1‐S6

## Data Availability

The sequence data generated in this study are readily available on GenBank with accession numbers MW885769 to MW885776, MW886319 to MW886322, and MW898152 to MW898213.

## APPENDIX 1

Details of samples used in the phylogenetic analysis, as well as in genetic distances

**Table:**

Genus	Species	Gene	Voucher No.	GenBank accession No.	Country/locality	Lat	Long	Publication
*Ansonia*	*longidigita*	16S	VUB 0666	FJ882796	Malaysia: Borneo	–	–	Bocxlaer et al. (2009)
*Ingerophrynus*	*divergens*	16S	VUB 0602	FJ882802	Malaysia: Borneo	–	–	Bocxlaer et al. (2009)
*Adenomus*	*kelaartii*	16S	VUB 0171	FJ882780	Sri Lanka	–	–	Bocxlaer et al. (2009)
*Pedostibes*	*tuberculosus*	16S	SDB 4691	FJ882793	India: Western Ghats	–	–	Bocxlaer et al. (2009)
*Bufotes*	*cf. surdus*	16S	ZMMSU A‐4027	FJ882810	Iran	–	–	Bocxlaer et al. (2009)
*Bufotes*	*cf. pewzowi*	16S	NP B‐4‐1	FJ882811	Uzbekistan	–	–	Bocxlaer et al. (2009)
*Bufotes*	*cf. variabilis*	16S	VUB 1813	FJ882812	Turkey	–	–	Bocxlaer et al. (2009)
*Bufotes*	*viridis*	16S	NP B‐2‐1	FJ882813	Greece	–	–	Bocxlaer et al. (2009)
*Xanthophryne*	*koynayensis*	16S	SDB 2004‐012	FJ882782	India: Western Ghats	–	–	Bocxlaer et al. (2009)
*Duttaphrynus*	*melanostictus*	16S	VUB 0052	FJ882791	India: Western Ghats	–	–	Bocxlaer et al. (2009)
*Duttaphrynus*	sp.	16S	SDB 927	FJ882792	India: Western Ghats	–	–	Bocxlaer et al. (2009)
*Duttaphrynus*	*brevirostris*	16S	SDB 4714	FJ882786	India: Western Ghats	–	–	Bocxlaer et al. (2009)
*Duttaphrynus*	*atukoralei*	16S	VUB 0101	FJ882835	Sri Lanka	–	–	Bocxlaer et al. (2009)
*Duttaphrynus*	*dhufarensis*	16S	CAS 227584	FJ882837	Oman	–	–	Bocxlaer et al. (2009)
*Duttaphrynus*	*hololius*	16S	SDB 4240	FJ882781	India	–	–	Bocxlaer et al. (2009)
*Duttaphrynus*	*parietalis*	16S	SDB 10100	FJ882784	India: Western Ghats	–	–	Bocxlaer et al. (2009)
*Duttaphrynus*	*scaber*	16S	SDB 532	FJ882785	India: Western Ghats	–	–	Bocxlaer et al. (2009)
*Duttaphrynus*	*stuarti*	16S	CAS 221485	FJ882788	Myanmar	–	–	Bocxlaer et al. (2009)
*Duttaphrynus*	*crocus*	16S	CAS 220193	FJ882789	Myanmar	–	–	Bocxlaer et al. (2009)
*Duttaphrynus*	*himalayanus*	16S	SDB4566	FJ882790	India	–	–	Bocxlaer et al. (2009)
*Duttaphrynus*	*himalayanus*	16S	DH3	KY000450	India: Uttarakhand	29.868236	79.310841	Bahuguna et al. (2017) Unpublished
*Duttaphrynus*	*melanostictus*	16S	DM2	KY000456	India: Uttarakhand	30.324483	78.102409	Bahuguna et al. (2017) Unpublished
*Duttaphrynus*	*melanostictus*	16S	KIZ‐95L001	AF160790	China: Yunnan Pingbian Co	22.790772	103.90988	Liu et al. (2000)
*Duttaphrynus*	*melanostictus*	16S	ROM 33162	AF160791	Vietnam: Gia Lia, Koh Rong	11.190175	103.86354	Liu et al. (2000)
*Duttaphrynus*	*melanostictus*	16S	1	EU071740	India: Pune, Maharashtra	18.576642	74.049723	Shouche and Ghate (2007) Unpublished
*Duttaphrynus*	sp.	16S	SDB 4594	FJ882839	India	–	–	Bocxlaer et al. (2009)
*Duttaphrynus*	*stomaticus*	16S	27	EU071742	India	–	–	Shouche and Ghate (2007) Unpublished
*Duttaphrynus*	*stomaticus*	16S	CCMB D02_B247	KT991344	India	–	–	Chandramouli et al. (2016)
*Duttaphrynus*	*stomaticus*	16S	SDB 4020	FJ882787	India: Western Ghats	–	–	Bocxlaer et al. (2009)
*Duttaphrynus*	*melanostictus*	16S	WLM:DM296164	MW885769	Pakistan: Kotli Sattian, Nalari	33.81554079	73.47496593	This study
*Duttaphrynus*	*melanostictus*	16S	WLM:DM303174	MW885770	Pakistan: Islamabad, Bani Gala	33.719659	73.164594	This study
*Duttaphrynus*	*stomaticus*	16S	WLM:DS48176	MW885771	Pakistan: Islamabad, Bani Gala	33.72553314	73.19205455	This study
*Duttaphrynus*	*stomaticus*	16S	WLM:DS2361610	MW885776	Pakistan: Taxila: Bahtar road	33.75005567	72.71150025	This study
*Duttaphrynus*	*stomaticus*	16S	WLM:DS46168	MW885774	Pakistan: Taxila: UET	33.75794448	72.82491694	This study
*Duttaphrynus*	*stomaticus*	16S	WLM:DS236168	MW885772	Pakistan: Rawalpindi: Chakri	33.47833084	72.93900073	This study
*Duttaphrynus*	*stomaticus*	16S	WLM:DS236169	MW885773	Pakistan: Kahuta: Baroothi	33.53938907	73.50488902	This study
*Duttaphrynus*	*stomaticus*	16S	WLM:DS46169	MW885775	Pakistan: Gujar Khan: Manghot	33.2755836	73.12263851	This study
*Uperodon*	*systoma*	16S	1151UpeSys	EF017960	India	–	–	Bocxlaer et al. (2006)
*Kaloula*	*pulchra*	16S	MBM‐USNMFS36482	MG935853	Myanmar: Kandawgyi National Gardens	21.9931	96.4713	Mulcahy et al. (2018)
*Microhyla*	*fissipes*	16S	KUHE32943	AB201185	China: Anhui, Huangshan	30.034532	118.0078	Matsui et al. (2005)
*Microhyla*	*mukhlesuri*	16S	SDBDU 2010.1332	MH549575	India: Mizoram	22.53	92.89	Garg et al. (2018)
*Microhyla*	*mukhlesuri*	16S	Not mentioned	JQ621935	Thailand	13.855145	100.76215	Gao and Fan (2012). Unpublished
*Microhyla*	*mukhlesuri*	16S	KIZHERP0138	JX678905	Vietnam	19.991547	104.13737	Li et al. (2012)
*Microhyla*	*mukhlesuri*	16S	TZ52	AF285202	Vietnam	17.650995	106.01649	Ziegler, T. (2000). Unpublished thesis
*Microhyla*	*mukhlesuri*	16S	16SMicrohyla_ornata	AF215373	Madagascar	−18.60814	46.61644	Vences (2000). Unpublished thesis
*Microhyla*	*chakrapanii*	16S	not preserved	MH807389	India: Andaman Islands	12.565535	92.80566	Garg et al. (2019)
*Microhyla*	*mymensinghensis*	16S	SDBDU 2015.2904	MH549590	India: West Bengal	22.43	88.38	Garg et al. (2018)
*Microhyla*	*rubra*	16S	SDBDU 2014.2829	MH807420	India: Tamil Nadu, Meenakshipuram	10.631822	76.86593	Garg et al. (2019)
*Microhyla*	*rubra*	16S	not preserved	AB201192	India; Karnataka	15.587753	75.863205	Matsui et al. (2005)
*Microhyla*	*taraiensis*	16S	JRK201525	KY655952	Nepal: Mechi	27.152954	87.886133	Khatiwada et al. (2017)
*Microhyla*	*taraiensis*	16S	JRK201527	KY655954	East Nepal	29.10608	82.246342	Khatiwada et al. (2017)
*Microhyla*	*taraiensis*	16S	JRK201526	KY655953	East Nepal	29.326619	81.916752	Khatiwada et al. (2017)
*Microhyla*	*ornata*	16S	SDBDU 2008.1720	MH549636	India: Tamil Nadu, Coimbatore	11.005298	76.942513	Garg et al. (2018)
*Microhyla*	*ornata*	16S	ZSIK‐A9119	AB201188	India: Karnataka, Dharwad	15.464739	74.98685	Matsui et al. (2005)
*Microhyla*	*ornata*	16S	RGCB15059	KP072794	India: Pulpally, Wayanad District, Kerala	11.7902	76.181561	Howlader et al. (2015a)
*Microhyla*	*ornata*	16S	SDBDU 2015.2898	MH549619	India: Andhra Pradesh Maredumilli	17.66	82.22	Garg et al. (2018)
*Microhyla*	*ornata*	16S	DZ 1432	MH807404	Sri Lanka: Kukulamalpotha	7.412246	81.159342	Garg et al. (2019)
*Microhyla*	*ornata*	16S	DZ 1427	MH807402	Sri Lanka: Makandura	7.323117	79.977009	Garg et al. (2019)
*Microhyla*	*ornata*	16S	SDBDU 2008.1958	MH549630	India: Tamil Nadu, Keeriparai	8.395153	77.412114	Garg et al. (2018)
*Microhyla*	*ornata*	16S	DZ 1104	MH807400	Sri Lanka: Puttalam	8.043706	79.835967	Garg et al. (2019)
*Microhyla*	*nilphamariensis*	16S	IABHU<JPN>:4213	LC090056	Bangladesh: Nilphamari, Barua, Berakhuti	25.886874	88.945658	Hasan et al. (2015)
*Microhyla*	*nilphamariensis*	16S	Morn‐Bd1	AB530537	Bangladesh: Dinajpur, Parbatipur	25.655386	88.912375	Hasan et al. (2012)
*Microhyla*	*nilphamariensis*	16S	Morn‐Bd3	AB530539	Bangladesh: Dinajpur, Parbatipur	25.653732	88.918973	Hasan et al. (2012)
*Microhyla*	*nilphamariensis*	16S	Morn‐Bd2	AB530538	Bangladesh: Dinajpur, Parbatipur	25.657774	88.916044	Hasan et al. (2012)
*Microhyla*	*nilphamariensis*	16S	ABHU<JPN>:4212	LC090055	Bangladesh: Nilphamari, Barua, Berakhuti	25.887231	88.947224	Hasan et al. (2015)
*Microhyla*	*nilphamariensis*	16S	MHLB00206	LC090057	Bangladesh: Nilphamari, Barua, Berakhuti	25.886102	88.944821	Hasan et al. (2015)
*Microhyla*	*nilphamariensis*	16S	SDBDU 2015.2905	MH549592	India: Assam, Barpeta, Mandia	26.268615	90.960971	Garg et al. (2018)
*Microhyla*	*nilphamariensis*	16S	MZH‐2362	KP072789	Bangladesh: Saidpur	25.781567	88.902439	Howlader et al. (2015a)
*Microhyla*	*nilphamariensis*	16S	ADWII_DT1	MH549616	India: Uttarakhand, Tuntowala	30.279222	78.004864	Garg et al. (2018)
*Microhyla*	*nilphamariensis*	16S	ADWII_M03	MH549618	India: Uttar Pradesh, Rajghat	28.241659	78.361208	Garg et al. (2018)
*Microhyla*	*nilphamariensis*	16S	MZH‐2363	KP072790	Bangladesh: Saidpur	25.79115	88.889736	Howlader et al. (2015a)
*Microhyla*	*nilphamariensis*	16S	M1_2_16SF‐A09.ab1	MN952999	Laterite Plateau of Western India	23.868377	72.611802	Mudke (2020). Unpublished
*Microhyla*	*nilphamariensis*	16S	M1_3_16SF‐B09.ab1	MN953000	Laterite Plateau of Western India	24.056953	72.810493	Mudke (2020). Unpublished
*Microhyla*	*nilphamariensis*	16S	M1_1_16SF‐H08.ab1	MN952998	Laterite Plateau of Western India	19.559477	73.761439	Mudke (2020). Unpublished
*Microhyla*	*nilphamariensis*	16S	SDBDU 2004.4507	MH549606	India: Maharashtra, Koyna	19.636949	73.170211	Garg et al. (2018)
*Microhyla*	*nilphamariensis*	16S	JRK201514	KY655939	Nepal: Jhuwani, district Chitwan, Narayani	27.591617	84.525397	Khatiwada et al. (2017)
*Microhyla*	*nilphamariensis*	16S	JRK201529	KY655951	Nepal: Hangdewa, Taplujung district, Mechi province	27.378704	87.700172	Khatiwada et al. (2017)
*Microhyla*	*nilphamariensis*	16S	JRK201501	KY655926	Nepal: Jhuwani, district Chitwan, Narayani province	27.594508	84.522865	Khatiwada et al. (2017)
*Microhyla*	*nilphamariensis*	16S	JRK201523	KY655948	Nepal: Jhuwani, district Chitwan, Narayani province	27.588042	84.529431	Khatiwada et al. (2017)
*Microhyla*	*nilphamariensis*	16S	JRK201518	KY655943	Nepal: Jhuwani, district Chitwan, Narayani province	27.58595	84.526341	Khatiwada et al. (2017)
*Microhyla*	*nilphamariensis*	16S	JRK201524	KY655949	Nepal: Jhuwani, district Chitwan, Narayani province	27.584894	84.529398	Khatiwada et al. (2017)
*Microhyla*	*nilphamariensis*	16S	WLM:MN236165	MW886319	Pakistan: Rawalpindi: Misrial	33.190561	72.808795	This study
*Microhyla*	*nilphamariensis*	16S	WLM:MN236163	MW886320	Pakistan: Rawalpindi, Chakri	33.51785089	72.97597999	This study
*Microhyla*	*nilphamariensis*	16S	WLM:MN46164	MW886321	Pakistan: Murree, Shangrila	33.856779	73.373913	This study
*Microhyla*	*nilphamariensis*	16S	WLM:MN236164	MW886322	Pakistan: Rawalpindi: Girja road	33.557083	72.995833	This study
*Rana*	*catesbeiana*	16S	NIBRAM0000100407	JQ815323	Korea: Gyeongsangnam‐do Jinju‐si Jeongchon‐myeon	–	–	Jeong et al. (2013)
*Rana*	*asiatica*	16S	Asia. R	AB058884	Russia: Kirghizia	–	–	Sumida et al. (2003)
*Quasipaa*	*shini*	16S	XJW‐LS‐001	KF199148	China: Longsheng, Guangxi	–	–	Zhang et al. (2018)
*Quasipaa*	*boulengeri*	16S	JFW‐TS‐002	KF199152	China: Tongshan, Hubei	–	–	Zhang et al. (2018)
*Quasipaa*	*exilispinosa*	16S	XJW‐WYS‐001	KF199151	China: Wuyishan, Fujian	–	–	Zhang et al. (2018)
*Quasipaa*	*jiulongensis*	16S	JLJW‐WYS‐002	KF199149	China: Wuyishan, Fujian	–	–	Zhang et al. (2018)
*Nanorana*	*cf. rarica*	16S	A1961/13_NME	MN012202	Central Himalaya	29.51	82.09	Hofmann et al. (2019)
*Nanorana*	*cf. rarica*	16S	A1970/13_NME	MN012203	Central Himalaya	29.51	82.09	Hofmann et al. (2019)
*Nanorana*	*cf. rarica*	16S	A2015/13_NME	MN012204	Central Himalaya	29.51	82.09	Hofmann et al. (2019)
*Nanorana*	*cf. rarica*	16S	A1960/13_NME	MN012207	Central Himalaya	29.36	82.2	Hofmann et al. (2019)
*Nanorana*	*cf. rarica*	16S	A2019/13_NME	MN012205	Central Himalaya	29.51	82.09	Hofmann et al. (2019)
*Nanorana*	*cf. rarica*	16S	A1965/13_NME	MN012206	Central Himalaya	29.513	82.092	Hofmann et al. (2019)
*Nanorana*	sp.	16S	R5_12_NME	MN012169	Central Himalaya	28.513	83.033	Hofmann et al. (2019)
*Nanorana*	sp.	16S	SH070556_NME	MN012172	Central Himalaya	28.622	83.662	Hofmann et al. (2019)
*Nanorana*	sp.	16S	Ne13_13_NME	MN012194	Central Himalaya	27.576	86.514	Hofmann et al. (2019)
*Nanorana*	sp.	16S	Ne1_13_NME	MN012195	Central Himalaya	27.689	86.731	Hofmann et al. (2019)
*Nanorana*	sp.	16S	Ne2_13_NME	MN012196	Central Himalaya	27.689	86.731	Hofmann et al. (2019)
*Nanorana*	sp.	16S	Ne9_13_NME	MN012197	Central Himalaya	27.671	86.765	Hofmann et al. (2019)
*Nanorana*	sp.	16S	SH080545_NME	MN012187	Central Himalaya	27.703	86.337	Hofmann et al. (2019)
*Nanorana*	sp.	16S	SH080593_NME	MN012180	Central Himalaya	27.686	86.252	Hofmann et al. (2019)
*Nanorana*	sp.	16S	R1_09_13_NME	MN012174	Central Himalaya	28.38	84.065	Hofmann et al. (2019)
*Nanorana*	sp.	16S	R2_09_13_NME	MN012175	Central Himalaya	28.38	84.065	Hofmann et al. (2019)
*Nanorana*	sp.	16S	SH070510_NME	MN012177	Central Himalaya	28.074	85.302	Hofmann et al. (2019)
*Nanorana*	sp.	16S	SH080551_NME	MN012190	Central Himalaya	27.703	86.337	Hofmann et al. (2019)
*Nanorana*	sp.	16S	SH080592_NME	MN012179	Central Himalaya	27.686	86.252	Hofmann et al. (2019)
*Nanorana*	sp.	16S	SH080594_NME	MN012181	Central Himalaya	27.686	86.252	Hofmann et al. (2019)
*Nanorana*	sp.	16S	SH080570_NME	MN012182	Central Himalaya	27.697	86.275	Hofmann et al. (2019)
*Nanorana*	sp.	16S	SH080571_NME	MN012183	Central Himalaya	27.697	86.275	Hofmann et al. (2019)
*Nanorana*	sp.	16S	SH080572_NME	MN012184	Central Himalaya	27.697	86.275	Hofmann et al. (2019)
*Nanorana*	sp.	16S	SH080553_NME	MN012185	Central Himalaya	27.718	86.311	Hofmann et al. (2019)
*Nanorana*	sp.	16S	SH080555_NME	MN012186	Central Himalaya	27.718	86.311	Hofmann et al. (2019)
*Nanorana*	sp.	16S	SH080546_NME	MN012188	Central Himalaya	27.703	86.337	Hofmann et al. (2019)
*Nanorana*	sp.	16S	SH080548_NME	MN012189	Central Himalaya	27.703	86.337	Hofmann et al. (2019)
*Nanorana*	sp.	16S	SH080552_NME	MN012191	Central Himalaya	27.703	86.337	Hofmann et al. (2019)
*Nanorana*	sp.	16S	SH080512_NME	MN012192	Central Himalaya	27.595	86.34	Hofmann et al. (2019)
*Nanorana*	sp.	16S	SH080591_NME	MN012178	Central Himalaya	27.686	86.252	Hofmann et al. (2019)
*Nanorana*	sp.	16S	SH080523_NME	MN012193	Central Himalaya	27.694	86.351	Hofmann et al. (2019)
*Nanorana*	*parkeri*	16S	SYNU‐1706031	MH315959	China	–	–	Qi et al. (2019). Unpublished
*Nanorana*	*parkeri*	16S	KizYP205	DQ118498	China	–	–	Hu et al. (2006). Unpublished
*Nanorana*	*parkeri*	16S	TP3_06_NME	MN012136	Transhimalaya and adjacent parts of the Tibetan Plateau	29.578	90.435	Hofmann et al. (2019)
*Nanorana*	*parkeri*	16S	TP7_06_NME	MN012155	Transhimalaya and adjacent parts of the Tibetan Plateau	31.166	92.061	Hofmann et al. (2019)
*Nanorana*	*parkeri*	16S	N7_06_NME	MN012126	Transhimalaya and adjacent parts of the Tibetan Plateau	29.589	90.214	Hofmann et al. (2019)
*Nanorana*	*parkeri*	16S	TP5_06_NME	MN012153	Transhimalaya and adjacent parts of the Tibetan Plateau	31.166	92.061	Hofmann et al. (2019)
*Nanorana*	*parkeri*	16S	CAS805L	MN012141	Transhimalaya and adjacent parts of the Tibetan Plateau	30.09	90.48	Hofmann et al. (2019)
*Nanorana*	*ventripunctata*	16S	KizYP200	DQ118502	China	–	–	Hu et al. (2006). Unpublished
*Nanorana*	*ventripunctata*	16S	KizYP201	DQ118503	China	–	–	Hu et al. (2006). Unpublished
*Nanorana*	*ventripunctata*	16S	SH050538_NME	MN012208	(sub) alpine parts of the eastern margin of the Tibetan Plateau	27.788	99.855	Hofmann et al. (2019)
*Nanorana*	*ventripunctata*	16S	SH050539_NME	MN012209	(sub) alpine parts of the eastern margin of the Tibetan Plateau	27.788	99.855	Hofmann et al. (2019)
*Nanorana*	*pleskei*	16S	KQ20_14_NME	MN012165	(sub) alpine parts of the eastern margin of the Tibetan Plateau	30.377	101.675	Hofmann et al. (2019)
*Nanorana*	*pleskei*	16S	KQ9_14_NME	MN012166	(sub) alpine parts of the eastern margin of the Tibetan Plateau	30.377	101.675	Hofmann et al. (2019)
*Nanorana*	*pleskei*	16S	KQ17_14_NME	MN012162	(sub) alpine parts of the eastern margin of the Tibetan Plateau	30.377	101.675	Hofmann et al. (2019)
*Nanorana*	*pleskei*	16S	CAS201	MN012167	(sub)alpine parts of the eastern margin of the Tibetan Plateau	33.467	102.75	Hofmann et al. (2019)
*Nanorana*	*pleskei*	16S	CAS202	MN012168	(sub)alpine parts of the eastern margin of the Tibetan Plateau	33.467	102.75	Hofmann et al. (2019)
*Nanorana*	*pleskei*	16S	KizYP203	DQ118504	China	–	–	Hu et al. (2006). Unpublished
*Nanorana*	*pleskei*	16S	KizYP204	DQ118505	China	–	–	Hu et al. (2006). Unpublished
*Nanorana*	*yunnanensis*	16S	‐	KF199150	China	–	–	Zhang and Yu (2017). Unpublished
*Nanorana*	*taihangnica*	16S	‐	KJ569109	China	–	–	Chen et al. (2015)
*Allopaa*	*hazarensis*	16S	WLM:AH265163	MW898152	Pakistan: Murree: Parhana	33.84322266	73.46941712	This study
*Allopaa*	*hazarensis*	16S	WLM:AH461611	MW898153	Pakistan: Murree, Bastal Mor (Shangrila Park)	33.8608605	73.37946601	This study
*Allopaa*	*hazarensis*	16S	WLM:AH305164	MW898154	Pakistan: Murree, Patriata road	33.86869227	73.4672	This study
*Allopaa*	*hazarensis*	16S	WLM:AH305168	MW898155	Pakistan: Murree: Patriata road	33.86869227	73.4672	This study
*Allopaa*	*hazarensis*	16S	WLM:AH265161	MW898156	Pakistan: Murree: Bell Garan	33.88477419	73.50041681	This study
*Allopaa*	*hazarensis*	16S	AH2651611	MW898157	Pakistan: Murree: Bell Garan	33.88477419	73.50041681	This study
*Allopaa*	*hazarensis*	16S	WLM:AH299171	MW898158	Pakistan: Murree: Murree expressway	33.84833345	73.42944468	This study
*Allopaa*	*hazarensis*	16S	WLM:AH299174	MW898159	Pakistan: Murree: Parhana	33.84324964	73.46955605	This study
*Allopaa*	*hazarensis*	16S	WLM:AH305167	MW898160	Pakistan: Murree: Aliyot	33.90124184	73.43383471	This study
*Allopaa*	*hazarensis*	16S	WLM:AH296163	MW898161	Pakistan: Murree: Mall road	33.9877782	73.49277785	This study
*Allopaa*	*hazarensis*	16S	WLM:AH305162	MW898162	Pakistan: Murree: Jhika Gali	33.91422267	73.4167499	This study
*Allopaa*	*hazarensis*	16S	WLM:AH28516t1	MW898163	Pakistan: Murree: Army dog center	33.91430535	73.39388838	This study
*Allopaa*	*hazarensis*	16S	WLM:AH305165	MW898164	Pakistan: Murree: Jhika Gali	33.91422267	73.39388838	This study
*Allopaa*	*hazarensis*	16S	WLM:AH299175	MW898165	Pakistan: Kotli Sattian: Kyonian	33.75205517	73.49188943	This study
*Allopaa*	*hazarensis*	16S	WLM:AH289174	MW898166	Pakistan: Kotli Sattian: New Koreana	33.82288846	73.52886145	This study
*Allopaa*	*hazarensis*	16S	WLM:AH299173	MW898167	Pakistan: Kotli Sattian: Kyonian	33.75205517	73.49188943	This study
*Allopaa*	*hazarensis*	16S	WLM:AH289173	MW898168	Pakistan: Kotli Sattian: Nalari	33.81372258	73.50108327	This study
*Allopaa*	*hazarensis*	16S	WLM:AH289175	MW898169	Pakistan: Kotli Sattian: Nalari	33.81372258	73.50108327	This study
*Allopaa*	*hazarensis*	16S	WLM:AH252171	MW898170	Pakistan: Murree: Chaka Begwal	33.797	73.37674988	This study
*Allopaa*	*hazarensis*	16S	WLM:AH296161	MW898171	Pakistan: Murree: Angoori	33.81458356	73.36883365	This study
*Allopaa*	*hazarensis*	16S	WLM:AH296162	MW898172	Pakistan: Murree: Angoori police station	33.79941651	73.35383292	This study
*Nanorana*	*cf. polunini*	16S	R3_09_13_NME	MN012087	Central Himalaya	28.38	84.065	Hofmann et al. (2019)
*Nanorana*	*cf. blanfordii*	16S	JS040534_NME	MN012071	Central Himalaya	27.617	87.233	Hofmann et al. (2019)
*Nanorana*	*cf. blanfordii*	16S	JS060520_NME	MN012073	Central Himalaya	27.173	87.421	Hofmann et al. (2019)
*Nanorana*	*cf. blanfordii*	16S	JS060515_NME	MN012074	Central Himalaya	27.214	87.463	Hofmann et al. (2019)
*Nanorana*	*cf. blanfordii*	16S	JS060508_NME	MN012075	Central Himalaya	27.413	87.734	Hofmann et al. (2019)
*Nanorana*	*cf. blanfordii*	16S	JS040529_NME	MN012067	Central Himalaya	27.617	87.233	Hofmann et al. (2019)
*Nanorana*	*cf. blanfordii*	16S	JS040532_NME	MN012069	Central Himalaya	27.617	87.233	Hofmann et al. (2019)
*Nanorana*	*cf. blanfordii*	16S	JS040531_NME	MN012068	Central Himalaya	27.617	87.233	Hofmann et al. (2019)
*Nanorana*	*cf. blanfordii*	16S	JS040535_NME	MN012072	Central Himalaya	27.617	87.233	Hofmann et al. (2019)
*Nanorana*	*cf. blanfordii*	16S	JS040533_NME	MN012070	Central Himalaya	27.617	87.233	Hofmann et al. (2019)
*Nanorana*	*cf. polunini*	16S	R15_12_NME	MN012082	Central Himalaya	28.502	83.129	Hofmann et al. (2019)
*Nanorana*	sp.	16S	2Pul_RAS	MN012200	NW Himalaya, (Himachal Pradesh)	31.996	77.448	Hofmann et al. (2019)
*Nanorana*	sp.	16S	782_RAS	MN012201	NW Himalaya, (Himachal Pradesh)	31.261	77.45	Hofmann et al. (2019)
*Nanorana*	sp.	16S	2Bhan_RAS	MN012199	NW Himalaya, (Himachal Pradesh)	32.873	75.858	Hofmann et al. (2019)
*Nanorana*	*vicina*	16S	WLM:NV28917	MW898173	Pakistan: Murree: Surasi	33.86869227	73.46719617	This study
*Nanorana*	*vicina*	16S	WLM:NV289171	MW898174	Pakistan: Murree: Bell Garan	33.88477419	73.50041681	This study
*Nanorana*	*vicina*	16S	WLM:NV299172	MW898175	Pakistan: Murree expressway	33.84833345	73.42944468	This study
*Nanorana*	*vicina*	16S	WLM:NV265166	MW898176	Pakistan: Murree: Mall road	33.9877782	73.49277785	This study
*Nanorana*	*vicina*	16S	WLM:NV289172	MW898177	Pakistan: Murree, Parhana	33.84324964	73.46955605	This study
*Nanorana*	*vicina*	16S	WLM:NV1310171	MW898178	Pakistan: Murree: Jhika Gali	33.91422267	73.4167499	This study
*Nanorana*	*vicina*	16S	WLM:NV26516t3	MW898179	Pakistan: Kotli Sattian: New Koreana	33.82288846	73.52886145	This study
*Nanorana*	*vicina*	16S	WLM:NV265164	MW898180	Pakistan: Kotli Sattian: Ratta Kas	33.72438924	73.45886097	This study
*Nanorana*	*vicina*	16S	WLM:NV265167	MW898181	Pakistan: Murree: Angoori (Police station)	33.79941651	73.35383292	This study
*Nanorana*	*cf. ercepeae*	16S	A2017/13_NME	MN012076	Central Himalaya	29.374	81.137	Hofmann et al. (2019)
*Nanorana*	*cf. ercepeae*	16S	A5_12_NME	MN012080	Central Himalaya	28.857	82.976	Hofmann et al. (2019)
*Nanorana*	*cf. rostandi*	16S	R17_12_NME	MN012101	Central Himalaya	28.519	83.264	Hofmann et al. (2019)
*Nanorana*	*cf. rostandi*	16S	SH070550_NME	MN012102	Central Himalaya	28.683	83.591	Hofmann et al. (2019)
*Nanorana*	sp.	16S	A1966/13_NME	MN012198	Central Himalaya (Chainpur Himal)	29.374	81.137	Hofmann et al. (2019)
*Nanorana*	*cf. polunini*	16S	SH070507_NME	MN012084	Central Himalaya	28.06	85.294	Hofmann et al. (2019)
*Nanorana*	*cf. polunini*	16S	SH070509_NME	MN012085	Central Himalaya	28.08	85.295	Hofmann et al. (2019)
*Nanorana*	*cf. polunini*	16S	SH070531_NME	MN012086	Central Himalaya	27.965	85.472	Hofmann et al. (2019)
*Nanorana*	*liebigii*	16S	A17_12_NME	MN012104	Central Himalaya	28.519	83.264	Hofmann et al. (2019)
*Nanorana*	*liebigii*	16S	SH070515_NME	MN012106	Central Himalaya	28.099	85.317	Hofmann et al. (2019)
*Nanorana*	*liebigii*	16S	SH080506_NME	MN012108	Central Himalaya	27.609	86.295	Hofmann et al. (2019)
*Nanorana*	*liebigii*	16S	Ne16_13_NME	MN012115	Central Himalaya	27.584	86.411	Hofmann et al. (2019)
*Nanorana*	*liebigii*	16S	JS040512_NME	MN012119	Central Himalaya	27.631	87.224	Hofmann et al. (2019)
*Nanorana*	*liebigii*	16S	JS060503_NME	MN012124	Central Himalaya	27.407	87.752	Hofmann et al. (2019)
*Nanorana*	*liebigii*	16S	JS060511_NME	MN012122	Central Himalaya	27.296	87.535	Hofmann et al. (2019)
*Nanorana*	*liebigii*	16S	KIZ‐RDXZL1	DQ118499	Central Himalaya	27.485	88.907	Hofmann et al. (2019)
*Nanorana*	*liebigii*	16S	2003.308	KR827956	Nepal: Pangum			Grosjean et al. (2015)
*Nanorana*	*liebigii*	16S	SH0805109_NME	MN012107	Central Himalaya	27.673	86.24	Hofmann et al. (2019)
*Nanorana*	*liebigii*	16S	Ne12_13_NME	MN012117	Central Himalaya	27.584	86.594	Hofmann et al. (2019)
*Nanorana*	*liebigii*	16S	Ne10_13_NME	MN012118	Central Himalaya	27.586	86.635	Hofmann et al. (2019)
*Sphaerotheca*	*pluvialis*	16S	‐	AF249042	Sri Lanka	–	–	Bossuyt and Milinkovitch (2000)
*Sphaerotheca*	*dobsonii*	16S	Sdob‐In	AB530608	India: Bajipe, Mangalore	12.9804	74.883618	Hasan et al. (2014)
*Sphaerotheca*	*dobsonii*	16S	16S‐dob	AB277305	India: Bajipe	12.992151	74.878932	Kotaki et al. (2008)
*Sphaerotheca*	*dobsonii*	16S	INHER Amphibia‐86	KY215970	India: Tamhini, Pune, Maharashtra	18.477	73.427	Padhye et al. (2017)
*Sphaerotheca*	*dobsonii*	16S	WILD‐16‐AMP‐651	KY215971	India: Devi Hasool, Maharashtra	16.742	73.432	Padhye et al. (2017)
*Sphaerotheca*	*breviceps*	16S	BNHS 6005	KY215977	India: Tranquebar (Tharangambadi), Tamil Nadu	11.062	79.813	Padhye et al. (2017)
*Sphaerotheca*	*breviceps*	16S	WILD‐16‐AMP‐645	KY215978	India: Tranquebar (Tharangambadi), Tamil Nadu	11.062	79.813	Padhye et al. (2017)
*Sphaerotheca*	*magadha*	16S	ZSI/WRC/2179	MK694738	India: Nawadih village, Koderma, Jharkhand	24.417985	85.468	Prasad et al. (2019)
*Sphaerotheca*	*breviceps*	16S	WILD‐16‐AMP‐647	KY215976	India: Maithon, Jharkhand	23.776	86.809	Padhye et al. (2017)
*Sphaerotheca*	*breviceps*	16S	BNHS 6006	KY215975	India: Maithon, Jharkhand	23.776	86.809	Padhye et al. (2017)
*Sphaerotheca*	*rolandae*	16S	Sptr	GU191122	India: Rajasthan	–	–	Sharma et al. (2009). Unpublished
*Sphaerotheca*	*pashchima*	16S	WILD‐16‐AMP‐644	KY215994	India: Maharashtra, Pune, Tamhini	18.477	73.427	Padhye et al. (2017)
*Sphaerotheca*	*pashchima*	16S	WILD‐16‐AMP‐642	KY215991	India: Maharashtra, Saswad‐Waghapur Road, Ambodi village	18.308	74.083	Padhye et al. (2017)
*Sphaerotheca*	*pashchima*	16S	ZSI‐WRC A/1549	KY215980	India: Karnataka, Yellapur‐Haliyal Road	15.16	74.759	Padhye et al. (2017)
*Sphaerotheca*	*pashchima*	16S	BNHS 6013	KY215983	India: Maharashtra, Raigad District, Kolad	18.404	73.321	Padhye et al. (2017)
*Sphaerotheca*	*pashchima*	16S	BNHS 6018	KY215988	India: Gujarat, Dang District, Waghai‐Ahwa road	20.709	73.709	Padhye et al. (2017)
*Sphaerotheca*	*pashchima*	16S	WILD‐16‐AMP‐641	KY215990	India: Maharashtra, Ahmednagar District, Karjat, near Rehekuri WLS	18.598	74.974	Padhye et al. (2017)
*Sphaerotheca*	*pashchima*	16S	BNHS 6017	KY215987	India: Maharashtra, Chinchli to Salher fort road	20.747	73.973	Padhye et al. (2017)
*Sphaerotheca*	*pashchima*	16S	WILD‐16‐AMP‐643	KY215993	India: Gujarat, Veghai Road, Ahwa‐Dang	20.764	73.676	Padhye et al. (2017)
*Sphaerotheca*	*pashchima*	16S	BNHS 6012	KY215981	India: Karnataka, Near Yellapur	14.98	74.731	Padhye et al. (2017)
*Sphaerotheca*	*pashchima*	16S	ZSI‐WRC A/1550	KY215982	India: Karnataka, Near Yellapur	14.98	74.731	Padhye et al. (2017)
*Sphaerotheca*	*pashchima*	16S	SbHR25HNBGU	KX815437	India	29.91889	78.12618	Chowdhary et al. (2017). Unpublished
*Sphaerotheca*	*pashchima*	16S	Sb31HNBGU	KX815435	India	30.28476	77.97365	Chowdhary et al. (2017). Unpublished
*Sphaerotheca*	*pashchima*	16S	SbSG17HNBGU	KX815440	India	30.22177	78.78434	Chowdhary et al. (2017). Unpublished
*Sphaerotheca*	*pashchima*	16S	WLM:SP46163	MW898191	Pakistan: Rawalpindi: Chakri	33.553389	73.015766	This study
*Sphaerotheca*	*pashchima*	16S	WLM:SP236166	MW898192	Pakistan: Islamabad: River Korang (Way to Angoori)	33.78342224	73.26690085	This study
*Sphaerotheca*	*pashchima*	16S	WLM:SP177161	MW898193	Pakistan: Kahuta: Bandhya	33.62661116	73.47552765	This study
*Sphaerotheca*	*pashchima*	16S	WLM:SP177162	MW898194	Pakistan: Rawalpindi: Adiala Road	33.50751564	73.03706598	This study
*Sphaerotheca*	*pashchima*	16S	SbJM1HNBGU	KX815439	India	30.55444	79.56635	Chowdhary et al. (2017). Unpublished
*Fejervarya*	*cancrivora*	16S	‐	AF346810	Indonesia: Java	–	–	Veith et al. (2001)
*Fejervarya*	*cancrivora*	16S	FC‐3	AB570273	Indonesia: Malang, East Java	–	–	Kurniawan et al. (2014)
*Fejervarya*	*limnocharis*	16S	TZ25	AF285211	North Vietnam: Vietnam			Ziegler (2000). Unpublished thesis
*Fejervarya*	*limnocharis*	16S	MVZ226347	EU979847	Vietnam: Tam Dao, Vinh Phu Prov.	21.406484	105.6417	Che et al. (2009)
*Fejervarya*	*limnocharis*	16S	FL‐1	AB570262	Indonesia: Padang, West Sumatra	−0.943817	100.41878	Kurniawan et al. (2014)
*Fejervarya*	*limnocharis*	16S	FL‐4	AB570265	Indonesia: Rokan Hilir, Riau, Sumatra	1.763345	100.75021	Kurniawan et al. (2014)
*Fejervarya*	*limnocharis*	16S	‐	JQ621940	China: Yunnan (south west china)	–	–	Gao and Fan (2012). Unpublished
*Fejervarya*	*limnocharis*	16S	1999.5721	KR827740	Vietnam: Lao Cai, Sapa	22.335302	103.84612	Grosjean et al. (2015)
*Fejervarya*	*limnocharis*	16S	Okin(2)	AB070734	Japan: Okinawa	26.388602	127.78929	Sumida et al. (2002)
*Fejervarya*	*limnocharis*	16S	16SF15	KU840567	China: Long Quan, Sichuan prov.	−0.716667	112.44245	Goutte et al. (2016)
*Fejervarya*	*limnocharis*	16S	H001	HQ226055	Taiwan	–	–	Chang and Liu (2010). Unpublished
*Fejervarya*	*limnocharis*	16S	H006	HQ226060	Taiwan	–	–	Chang and Liu (2010). Unpublished
*Minervarya*	*rufescens*	16S	SDBDU 2015.2882	KY447323	India: Pozhuthana, Kerala	11.590328	76.02067	Garg and Biju (2017)
*Minervarya*	*greenei*	16S	‐	AB488891	Sri Lanka: Hakgala	6.918789	80.831133	Kotaki et al. (2010)
*Minervarya*	*kudremukhensis*	16S	‐	AB488898	India: Kudremukh (Karnataka)	13.222713	75.250694	Kotaki et al. (2010)
*Minervarya*	*sahyadris*	16S	Fsah‐In2	AB530605	India: Aralam (Kerala)	11.970321	75.800625	Hasan et al. (2014)
*Minervarya*	*caperata*	16S	‐	AB488894	India: Mudigere (Karnataka)	13.132451	75.640472	Kotaki et al. (2010)
*Minervarya*	*asmati*	16S	FaCSE_17	KP849815	Bangladesh: Dhaka	23.742528	90.453325	Howlader et al. (2016)
*Minervarya*	*asmati*	16S	HJZG‐04	MG010390	Bangladesh	–	–	Jahan et al. (2018). Unpublished
*Minervarya*	*granosa*	16S	BNHS 4652	AB355839	India: Western Ghats, Madikeri	10.246409	76.827714	Kuramoto et al. (2007)
*Minervarya*	*granosa*	16S	‐	AB488895	India: Mudigere (Karnataka)	13.131135	75.64207	Kotaki et al. (2010)
*Minervarya*	*syhadrensis*	16S	‐	AB488892	Sri Lanka: Hakgala	6.918789	80.831063	Kotaki et al. (2010)
*Minervarya*	*syhadrensis*	16S	WHT2665	AY141843	Sri Lanka	–	–	Meegaskumbura et al. (2002)
*Minervarya*	*pierrei*	16S	FSP 04	MK635483	Bangladesh	–	–	Jahan et al. (2019). Unpublished
*Minervarya*	*pierrei*	16S	F1DSE_14	KP849816	Bangladesh	–	–	Howlader et al. (2016)
*Minervarya*	*pierrei*	16S	Fsp.S‐Bd1	AB530509	Bangladesh: Mymensingh, Char Nilokhia	24.08494	90.8911	Hasan et al. (2012)
*Minervarya*	*pierrei*	16S	FSP 02	MK635481	Bangladesh	–	–	Jahan et al. (2019). Unpublished
*Minervarya*	*pierrei*	16S	‐	AB488888	Nepal: Chitwan	27.533841	84.436593	Kotaki et al. (2010)
*Minervarya*	*syhadrensis*	16S	IN023	AY882955	India: Kolkata: West Bengal	22.571807	88.427181	Tandon et al. (2005). Unpublished.
*Minervarya*	*pierrei*	16S	Fsp.S‐Bd2	AB530510	Bangladesh: Cox' s Bazar, Laboni point	21.438741	92.016288	Hasan et al. (2012)
*Minervarya*	*pierrei*	16S	WLM:MP48171	MW898182	Pakistan: Taxila: Margalla Hills National Park	33.731587	72.94793	This study
*Minervarya*	*pierrei*	16S	WLM:MP48173	MW898183	Pakistan: Gujar Khan: Susral	33.15186109	73.24997274	This study
*Minervarya*	*pierrei*	16S	WLM:MP48174	MW898184	Pakistan: Gujar Khan: Morha Phool	33.16852748	73.08461077	This study
*Minervarya*	*pierrei*	16S	WLM:MP48172	MW898185	Pakistan: Islamabad: Korang river	33.719977	73.147451	This study
*Minervarya*	*pierrei*	16S	WLM:MP48175	MW898186	Pakistan: Islamabad: Korang river	33.719977	73.147451	This study
*Minervarya*	*pierrei*	16S	WLM:MP236167	MW898187	Pakistan: Rawalpindi: Kalyal Sharef	33.53288389	73.05085836	This study
*Minervarya*	*syhadrensis*	16S	IN021	AY882953	India: Kolkata: West Bengal	22.630128	88.418941	Tandon et al. (2005). Unpublished.
*Minervarya*	*syhadrensis*	16S	IN016	AY882948	India: Kolkata: West Bengal	22.573076	88.442974	Tandon et al. (2005). Unpublished.
*Hoplobatrachus*	*rugulose*	16S	Chin‐Chacho‐3663‐16S	AB636617	Thailand: Chachoengsao	13.696414	101.64166	Alam et al. (2012)
*Hoplobatrachus*	*rugulose*	16S	Chin‐Chacho‐3911‐16S	AB636615	Thailand: Chachoengsao	13.746443	101.54347	Alam et al. (2012)
*Hoplobatrachus*	*tigerinus*	16S	31	AB167944	India: Mangalore, Padil	12.872854	74.888555	Kurabayashi et al. (2005)
*Hoplobatrachus*	*tigerinus*	16S	30	AB167943	India: Mangalore, Padil	12.869214	74.882375	Kurabayashi et al. (2005)
*Hoplobatrachus*	*tigerinus*	16S	Htig‐In	AB530600	India: Padil	12.867122	74.882085	Hasan et al. (2014)
*Hoplobatrachus*	*tigerinus*	16S	RGCB 5794	KU179090	India: Kerala	9.797816	76.895336	Anoop and George (2016). Unpublished
*Hoplobatrachus*	*tigerinus*	16S	RGCB 5060	KU179088	India: Kerala	9.803303	76.912811	Anoop and George (2016). Unpublished
*Hoplobatrachus*	*tigerinus*	16S	RGCB 5731	KU179089	India: Kerala	9.797383	76.915643	Anoop and George (2016). Unpublished
*Hoplobatrachus*	*tigerinus*	16S	tig‐baji‐A	AB290412	India: Bajipe	12.962778	74.890833	Alam et al. (2008)
*Hoplobatrachus*	*tigerinus*	16S	tig‐padi‐A	AB272594	India: Padil	12.869167	74.8825	Alam et al. (2008)
*Hoplobatrachus*	*tigerinus*	16S	WLM:HT46162	MW898188	Pakistan: Islamabad: Naval anchorage	33.568652	73.183754	This study
*Hoplobatrachus*	*tigerinus*	16S	WLM:HT236161	MW898189	Pakistan: Kallar Syedan: Bhyakrial	33.4835282	73.35569476	This study
*Hoplobatrachus*	*tigerinus*	16S	WLM:HT48177	MW898190	Pakistan: Rawalpindi: Adiala Road	33.482533	73.005784	This study
*Hoplobatrachus*	*tigerinus*	16S	IABHU<JPN>:4001	AB671183	Bangladesh: Mymensingh	24.745336	90.404392	Hasan et al. (2012)
*Hoplobatrachus*	*tigerinus*	16S	Tig‐Control‐3920‐16S	AB636619	Bangladesh: Mymensingh, BAU Campus	24.724225	90.428289	Alam et al. (2012)
*Hoplobatrachus*	*tigerinus*	16S	Htig‐Bd1	AB530500	Bangladesh: Mymensingh, BAU Campus	24.45	90.24	Hasan et al. (2012)
*Hoplobatrachus*	*tigerinus*	16S	IABHU<JPN>:4000	AB671182	Bangladesh: Mymensingh	24.745024	90.404563	Hasan et al. (2012)
*Hoplobatrachus*	*tigerinus*	16S	tig‐jaga‐B	AB272590	Bangladesh: Jagannathganj	24.75	89.816667	Alam et al. (2008)
*Hoplobatrachus*	*tigerinus*	16S	tig‐sylh‐A	AB272588	Bangladesh: Sylhet	24.92	92	Alam et al. (2008)
*Hoplobatrachus*	*tigerinus*	16S	tig‐BAUC‐B	AB272584	Bangladesh: Mymensingh, BAU Campus	24.747222	90.406667	Alam et al. (2008)
*Hoplobatrachus*	*tigerinus*	16S	tig‐BAUC‐A	AB272583	Bangladesh: Mymensingh, BAU Campus	24.738321	90.401817	Alam et al. (2008)
*Euphlyctis*	*aloysii*	16S	BNHS5995	KU870382	India: Karnataka	13.3728	74.8016	Priti et al. (2016)
*Euphlyctis*	*hexadactylus*	16S	hex‐mudi‐A	AB272608	India: Mudigere, Karnataka	12.87	74.92	Alam et al. (2008)
*Euphlyctis*	*hexadactylus*	16S	hex‐adya‐B	AB272607	India: Adyar, Karnataka	13.134444	75.641111	Alam et al. (2008)
*Euphlyctis*	*karaavali*	16S	BNHS5986	KU870373	India: Uttara Kannada, Karnataka	14.5512	74.3378	Priti et al. (2016)
*Euphlyctis*	*ehrenbergi*	16S	MNHN 2000.649	AY014367	Yemen	–	–	Kosuch et al. (2001)
*Euphlyctis*	*mudigere*	16S	Emud‐In	AB530599	India: Mudigere	13.142503	75.646705	Hasan et al. (2014)
*Euphlyctis*	*cyanophlyctis*	16S	RGCB 79	KU179080	India: Kerala	10.046289	77.003533	Anoop and George (2016). Unpublished
*Euphlyctis*	*cyanophlyctis*	16S	‐	EU523739	India: Kerala	10.026056	77.07216	Liya et al. (2008). Unpublished
*Euphlyctis*	*cyanophlyctis*	16S	RGCB 1018	KU179079	India: Nagapattinam, Tamil Nadu	10.780117	79.83786	Anoop and George (2016). Unpublished
*Euphlyctis*	*mudigere*	16S	‐	AF249053	India: Mudigere	12.904912	74.85309	Bossuyt and Milinkovitch (2000)
*Euphlyctis*	*cyanophlyctis*	16S	MNHN 2000.650	AY014366	India: Cochin	–	–	Kosuch et al. (2001)
*Euphlyctis*	*cyanophlyctis*	16S	cya‐padi‐B	AB272604	India: Padil	12.869167	74.8825	Alam et al. (2008)
*Euphlyctis*	*cyanophlyctis*	16S	030523–03	AB167938	India: Western Ghats, Madikeri	12.427069	75.731093	Kurabayashi et al. (2005)
*Euphlyctis*	*cyanophlyctis*	16S	‐	AB488901	India: Mangalore	12.904912	74.85309	Kotaki et al. (2010)
*Euphlyctis*	*cyanophlyctis*	16S	Ecya‐In	AB530596	India: Madikeri	12.429919	75.73281	Hasan et al. (2014)
*Euphlyctis*	*kalasgramensis*	16S	ZMUVAS5	MK920114	Pakistan: Pattoki, Kasur	31.044	73.87492	Ali et al. (2020)
*Euphlyctis*	*kalasgramensis*	16S	ZMUVAS1	MK881165	Pakistan: Pattoki, Kasur	31.044	73.8749	Ali et al. (2020)
*Euphlyctis*	*kalasgramensis*	16S	cya‐assa‐A	AB290420	India: Assam	26.165556	92.841389	Alam et al. (2008)
*Euphlyctis*	*kalasgramensis*	16S	cya‐BAUC‐A	AB272601	Bangladesh: Mymensingh, BAU Campus	24.747222	90.406667	Alam et al. (2008)
*Euphlyctis*	*kalasgramensis*	16S	Ecya‐Bd3	AB530496	Bangladesh: Cox's Bazar, Laboni point	21.41	91.98	Hasan et al. (2012)
*Euphlyctis*	*kalasgramensis*	16S	Ecya‐Bd4	AB530497	Bangladesh: Cox's Bazar, Laboni point	21.41	91.98	Hasan et al. (2012)
*Euphlyctis*	*kalasgramensis*	16S	Ecya‐Bd2	AB530495	Bangladesh: Mymensingh, Char Nilokhia	21.45	90.24	Hasan et al. (2012)
*Euphlyctis*	*kalasgramensis*	16S	ESP 02	MK635488	Bangladesh: Kalashram	22.760392	90.319291	Jahan et al. (2019). Unpublished
*Euphlyctis*	*kalasgramensis*	16S	HJZG‐03	MG010388	Bangladesh: Barisal	22.696171	90.355864	Jahan et al. (2019). Unpublished
*Euphlyctis*	*kalasgramensis*	16S	MZH‐3381	KP091868	Bangladesh: Kalashram	22.760392	90.317961	Howlader et al. (2015b)
*Euphlyctis*	*kalasgramensis*	16S	PUCZM/IX/SL64	MH087080	India: Mizoram	23.759	92.799	Lalronunga et al. (2019) Unpublished
*Euphlyctis*	*kalasgramensis*	16S	PUCZM/IX/SL613	MH087081	India: Mizoram	23.712	92.662	Lalronunga et al. (2019) Unpublished
*Euphlyctis*	*kalasgramensis*	16S	PUCZM/IX/SL62	MH087078	India: Mizoram	23.759	92.799	Lalronunga et al. (2019) Unpublished
*Euphlyctis*	*kalasgramensis*	16S	PUCZM/IX/SL63	MH087079	India: Mizoram	23.759	92.799	Lalronunga et al. (2019) Unpublished
*Euphlyctis*	*kalasgramensis*	16S	MZH‐3383	KP091856	Bangladesh: Barisal	22.694206	90.351395	Howlader et al. (2015b)
*Euphlyctis*	*cyanophlyctis*	16S	F1	KF992815	Iran: Tiran	32.701346	51.153867	Khajeh et al. (2014)
*Euphlyctis*	*cyanophlyctis*	16S	A1	KF992800	Iran: Apatan	27.350647	62.098627	Khajeh et al. (2014)
*Euphlyctis*	*kalasgramensis*	16S	WLM:EK303173	MW898195	Pakistan: Taxila: Village Khurram	33.7345002	72.86511099	This study
*Euphlyctis*	*kalasgramensis*	16S	WLM:EK48179	MW898196	Pakistan: Islamabad: Shahdara	33.78513863	73.17800038	This study
*Euphlyctis*	*kalasgramensis*	16S	WLM:EK48178	MW898197	Pakistan: Islamabad: Shahdara	33.78513863	73.17800038	This study
*Euphlyctis*	*kalasgramensis*	16S	WLM:EK481710	MW898198	Pakistan: Islamabad: Shahdara	33.78513863	73.17800038	This study
*Euphlyctis*	*kalasgramensis*	16S	WLM:EK236162	MW898199	Pakistan: Gujar Khan: Doltana	33.19633336	73.26858302	This study
*Euphlyctis*	*kalasgramensis*	16S	WLM:EK10917	MW898200	Pakistan: Murree: Manga	33.798224	73.294917	This study
*Euphlyctis*	*kalasgramensis*	16S	WLM:EK26516t6	MW898201	Pakistan: Islamabad: Talhar	33.770179	73.049072	This study
*Euphlyctis*	*kalasgramensis*	16S	WLM:EK265168	MW898202	Pakistan: Islamabad: Talhar	33.770179	73.049072	This study
*Euphlyctis*	*kalasgramensis*	16S	WLM:EK2010175	MW898203	Pakistan: Kahuta: Panjpeer	33.65313917	73.5337495	This study
*Euphlyctis*	*kalasgramensis*	16S	WLM:EK2010173	MW898204	Pakistan: Kahuta: Panjpeer	33.65313917	73.5337495	This study
*Euphlyctis*	*kalasgramensis*	16S	WLM:EK2010174	MW898205	Pakistan: Kahuta: Panjpeer	33.65313917	73.5337495	This study
*Euphlyctis*	*kalasgramensis*	16S	WLM:EK2010176	MW898206	Pakistan: Kahuta: Panjpeer	33.645182	73.460291	This study
*Euphlyctis*	*kalasgramensis*	16S	WLM:EK2010172	MW898207	Pakistan: Kahuta: Panjpeer	33.645182	73.460292	This study
*Euphlyctis*	*kalasgramensis*	16S	WLM:EK46161	MW898208	Pakistan: Kotli Sattian: Near Kahuta Checkpost	33.75554159	73.44597954	This study
*Euphlyctis*	*kalasgramensis*	16S	WLM:EK461612	MW898209	Pakistan: Rawalpindi: Soan river	33.436292	72.99796	This study
*Euphlyctis*	*kalasgramensis*	16S	WLM:EK252173	MW898210	Pakistan: Islamabad: Bani Gala	33.711083	73.161416	This study
*Euphlyctis*	*kalasgramensis*	16S	WLM:EK252172	MW898211	Pakistan: Islamabad: Bani Gala	33.711083	73.161416	This study
*Euphlyctis*	*kalasgramensis*	16S	WLM:EK305161	MW898212	Pakistan: Rawalpindi: Choanthra	33.352238	72.775779	This study
*Euphlyctis*	*kalasgramensis*	16S	WLM:EK31317t	MW898213	Pakistan: Rawalpindi: Udhual	33.312832	72.912557	This study

## APPENDIX 2

Primers used in the present study for PCR amplification

**Table:**

Gene	Primer	Sequence 5′‐3′	bp	Reference
16S	16SAR 16SBR	5′‐CGCCTGTTTAYCAAAAACAT‐3′ 5′‐CCGGTYTGAACTCAGATCAYGT‐3′	550	Palumbi (1996)
16S	16SC 16SD	5′‐GTRGGCCTAAAAGCAGCCAC‐3′ 5′‐CTCCGGTCTGAACTCAGATCACGTAG‐3′	950	Cannatella et al. (1998)

## APPENDIX 3

Bayesian inference analysis based on the 16S rRNA, of genus *Duttaphrynus*, Family Bufonidae. Posterior probability values are indicated near each node. Sequences generated in the present study are given in red.

## APPENDIX 4

Bayesian inference analysis based on the 16S rRNA, of genus *Microhyla*, Family Microhylidae. Posterior probability values are indicated near each node. Sequences generated in the present study are given in red.

## APPENDIX 5

Bayesian inference analysis based on the 16S rRNA, of genus *Nanorana* and *Allopaa*. Posterior probability values are indicated near each node. Sequences generated in the present study are given in red.

## APPENDIX 6

Bayesian inference analysis based on the 16S rRNA, of genus *Nanorana*. Posterior probability values are indicated near each node. Sequences generated in the present study are given in red.

## APPENDIX 7

Bayesian inference analysis based on the 16S rRNA, of genus *Sphaerotheca*, *Fejervarya,* and *Minervarya*. Posterior probability values are indicated near each node. Sequences generated in the present study are given in red.

## APPENDIX 8

Bayesian inference analysis based on the 16S rRNA, of genus *Hoplobatrachus* and *Euphlyctis*. Posterior probability values are indicated near each node. Sequences generated in the present study are given in red.
