# Supplementary material for: An insight into molecular taxonomy of bufonids, microhylids, and dicroglossid frogs: First genetic records from Pakistan
Source: Ecol Evol. 2021 Sep 28;11(20):14175–216. doi: 10.1002/ece3.8134 (PMC8525160; doi:10.1002/ece3.8134)
Supplement: Supplementary file 1 — Tables S1‐S6 [file ECE3-11-14175-s001.docx]

Table S1*: Uncorrected p distance* between groups of Family Bufonidae

| *Adenomus kelaartii* |  | 0.015 | 0.016 | 0.015 | 0.015 | 0.016 | 0.017 | 0.017 | 0.017 | 0.015 | 0.017 | 0.016 | 0.016 | 0.017 | 0.017 | 0.018 | 0.017 | 0.017 | 0.015 | 0.017 | 0.016 | 0.017 |
| --- | --- | --- | --- | --- | --- | --- | --- | --- | --- | --- | --- | --- | --- | --- | --- | --- | --- | --- | --- | --- | --- | --- |
| *Bufotes cf pewzowi* | 0.090 |  | 0.015 | 0.009 | 0.008 | 0.014 | 0.016 | 0.015 | 0.015 | 0.014 | 0.015 | 0.015 | 0.014 | 0.015 | 0.015 | 0.015 | 0.016 | 0.015 | 0.014 | 0.016 | 0.015 | 0.014 |
| *Bufotes cf surdus* | 0.118 | 0.090 |  | 0.015 | 0.015 | 0.015 | 0.016 | 0.016 | 0.017 | 0.015 | 0.016 | 0.015 | 0.016 | 0.017 | 0.016 | 0.017 | 0.016 | 0.017 | 0.015 | 0.016 | 0.017 | 0.015 |
| *Bufotes cf variabilis* | 0.096 | 0.028 | 0.090 |  | 0.006 | 0.015 | 0.015 | 0.015 | 0.015 | 0.014 | 0.015 | 0.015 | 0.014 | 0.015 | 0.016 | 0.016 | 0.015 | 0.016 | 0.014 | 0.015 | 0.015 | 0.014 |
| *Bufotes viridis* | 0.090 | 0.022 | 0.087 | 0.014 |  | 0.015 | 0.014 | 0.015 | 0.014 | 0.014 | 0.014 | 0.014 | 0.014 | 0.015 | 0.015 | 0.016 | 0.015 | 0.015 | 0.014 | 0.015 | 0.015 | 0.014 |
| *Duttaphrynus stomaticus* 2 | 0.118 | 0.087 | 0.112 | 0.101 | 0.093 |  | 0.016 | 0.015 | 0.016 | 0.011 | 0.015 | 0.013 | 0.015 | 0.016 | 0.015 | 0.015 | 0.016 | 0.015 | 0.009 | 0.016 | 0.015 | 0.015 |
| *Duttaphrynus atukoralei* | 0.126 | 0.098 | 0.112 | 0.096 | 0.081 | 0.112 |  | 0.013 | 0.014 | 0.015 | 0.013 | 0.016 | 0.014 | 0.013 | 0.014 | 0.013 | 0.009 | 0.013 | 0.015 | 0.014 | 0.016 | 0.016 |
| *Duttaphrynus brevirostris* | 0.124 | 0.093 | 0.112 | 0.093 | 0.090 | 0.104 | 0.065 |  | 0.012 | 0.014 | 0.012 | 0.015 | 0.010 | 0.011 | 0.010 | 0.010 | 0.014 | 0.009 | 0.014 | 0.015 | 0.017 | 0.016 |
| *Duttaphrynus crocus* | 0.118 | 0.093 | 0.121 | 0.098 | 0.084 | 0.112 | 0.081 | 0.073 |  | 0.016 | 0.013 | 0.016 | 0.013 | 0.014 | 0.012 | 0.013 | 0.014 | 0.013 | 0.016 | 0.015 | 0.016 | 0.015 |
| *Duttaphrynus dhufarensis* | 0.107 | 0.079 | 0.093 | 0.081 | 0.079 | 0.056 | 0.101 | 0.087 | 0.101 |  | 0.015 | 0.014 | 0.015 | 0.015 | 0.015 | 0.014 | 0.016 | 0.015 | 0.010 | 0.016 | 0.015 | 0.015 |
| *Duttaphrynus himalayanus* | 0.126 | 0.096 | 0.108 | 0.096 | 0.087 | 0.112 | 0.070 | 0.062 | 0.079 | 0.103 |  | 0.016 | 0.010 | 0.011 | 0.013 | 0.012 | 0.014 | 0.011 | 0.015 | 0.014 | 0.015 | 0.015 |
| *Duttaphrynus hololius* | 0.112 | 0.087 | 0.107 | 0.096 | 0.084 | 0.073 | 0.101 | 0.104 | 0.107 | 0.070 | 0.115 |  | 0.015 | 0.016 | 0.015 | 0.016 | 0.016 | 0.015 | 0.012 | 0.016 | 0.016 | 0.015 |
| *Duttaphrynus melanostictus* 1 | 0.117 | 0.093 | 0.114 | 0.088 | 0.087 | 0.110 | 0.074 | 0.049 | 0.072 | 0.096 | 0.055 | 0.097 |  | 0.007 | 0.012 | 0.012 | 0.014 | 0.011 | 0.014 | 0.015 | 0.015 | 0.015 |
| *Duttaphrynus melanostictus* 2 | 0.131 | 0.099 | 0.120 | 0.098 | 0.097 | 0.116 | 0.066 | 0.047 | 0.078 | 0.103 | 0.056 | 0.111 | 0.027 |  | 0.012 | 0.011 | 0.014 | 0.011 | 0.015 | 0.015 | 0.016 | 0.016 |
| *Duttaphrynus melanostictus* 3 | 0.129 | 0.093 | 0.118 | 0.104 | 0.096 | 0.107 | 0.073 | 0.039 | 0.065 | 0.096 | 0.070 | 0.096 | 0.060 | 0.057 |  | 0.010 | 0.014 | 0.009 | 0.014 | 0.015 | 0.017 | 0.016 |
| *Duttaphrynus parietalis* | 0.138 | 0.101 | 0.121 | 0.104 | 0.101 | 0.110 | 0.065 | 0.039 | 0.079 | 0.093 | 0.070 | 0.107 | 0.058 | 0.056 | 0.037 |  | 0.014 | 0.009 | 0.015 | 0.015 | 0.016 | 0.016 |
| *Duttaphrynus scaber* | 0.135 | 0.107 | 0.110 | 0.104 | 0.090 | 0.126 | 0.031 | 0.081 | 0.090 | 0.115 | 0.083 | 0.107 | 0.086 | 0.086 | 0.081 | 0.081 |  | 0.014 | 0.015 | 0.015 | 0.016 | 0.016 |
| *Duttaphrynus sp* | 0.132 | 0.104 | 0.121 | 0.110 | 0.101 | 0.115 | 0.070 | 0.031 | 0.073 | 0.107 | 0.063 | 0.101 | 0.052 | 0.054 | 0.034 | 0.037 | 0.081 |  | 0.015 | 0.015 | 0.016 | 0.016 |
| *Duttaphrynus stomaticus* 1 | 0.104 | 0.076 | 0.098 | 0.079 | 0.081 | 0.039 | 0.101 | 0.087 | 0.101 | 0.039 | 0.104 | 0.065 | 0.096 | 0.103 | 0.096 | 0.096 | 0.110 | 0.107 |  | 0.016 | 0.014 | 0.014 |
| *Duttaphrynus stuarti* | 0.121 | 0.096 | 0.104 | 0.093 | 0.087 | 0.112 | 0.087 | 0.096 | 0.087 | 0.101 | 0.083 | 0.101 | 0.088 | 0.095 | 0.090 | 0.096 | 0.098 | 0.096 | 0.104 |  | 0.016 | 0.014 |
| *Pedostibes tuberculosus* | 0.098 | 0.087 | 0.110 | 0.081 | 0.081 | 0.096 | 0.101 | 0.107 | 0.096 | 0.084 | 0.104 | 0.093 | 0.098 | 0.110 | 0.107 | 0.104 | 0.115 | 0.107 | 0.081 | 0.096 |  | 0.014 |
| *Xanthophryne koynayensis* | 0.110 | 0.081 | 0.101 | 0.079 | 0.076 | 0.098 | 0.107 | 0.104 | 0.093 | 0.087 | 0.100 | 0.098 | 0.098 | 0.112 | 0.101 | 0.101 | 0.112 | 0.107 | 0.084 | 0.087 | 0.079 |  |

Table S2: *Uncorrected p distance* within groups of Family Bufonidae

| *Adenomus kelaartii* | n/c | n/c |
| --- | --- | --- |
| *Bufotes cf pewzowi* | n/c | n/c |
| *Bufotes cf variabilis* | n/c | n/c |
| *Bufotes viridis* | n/c | n/c |
| *Xanthophryne koynayensis* | n/c | n/c |
| *Duttaphrynus dhufarensis* | n/c | n/c |
| *Duttaphrynus stomaticus* 2 | 0.017 | 0.006 |
| *Duttaphrynus stomaticus* 1 | 0.001 | 0.001 |
| *Duttaphrynus hololius* | n/c | n/c |
| *Pedostibes tuberculosus* | n/c | n/c |
| *Duttaphrynus melanostictus* 3 | n/c | n/c |
| *Duttaphrynus sp* | n/c | n/c |
| *Duttaphrynus melanostictus* 2 | 0.006 | 0.003 |
| *Duttaphrynus melanostictus* 1 | 0.014 | 0.006 |
| *Duttaphrynus brevirostris* | n/c | n/c |
| *Duttaphrynus parietalis* | n/c | n/c |
| *Duttaphrynus himalayanus* | 0.028 | 0.009 |
| *Duttaphrynus crocus* | n/c | n/c |
| *Duttaphrynus atukoralei* | n/c | n/c |
| *Duttaphrynus scaber* | n/c | n/c |
| *Duttaphrynus stuarti* | n/c | n/c |
| *Bufotes cf surdus* | n/c | n/c |

Table S3: *Uncorrected p distance* between Group of Family Microhylidae

|  |  | 0.01 | 0.01 | 0.01 | 0.01 | 0.01 | 0.01 | 0.01 | 0.01 |
| --- | --- | --- | --- | --- | --- | --- | --- | --- | --- |
| *Microhyla chakrapanii* | 0.10 |  | 0.01 | 0.01 | 0.01 | 0.01 | 0.01 | 0.02 | 0.02 |
| *Microhyla fissipes* | 0.10 | 0.04 |  | 0.01 | 0.01 | 0.01 | 0.01 | 0.01 | 0.01 |
| *Microhyla mukhlesuri* 1 | 0.10 | 0.05 | 0.03 |  | 0.01 | 0.01 | 0.01 | 0.01 | 0.01 |
| *Microhyla mymensinghensis* | 0.09 | 0.03 | 0.03 | 0.04 |  | 0.01 | 0.01 | 0.01 | 0.01 |
| *Microhyla nilphamariensis* | 0.05 | 0.10 | 0.10 | 0.09 | 0.09 |  | 0.01 | 0.01 | 0.01 |
| *Microhyla mukhlesuri* 2 | 0.10 | 0.05 | 0.02 | 0.02 | 0.04 | 0.09 |  | 0.01 | 0.01 |
| *Microhyla rubra* | 0.09 | 0.11 | 0.10 | 0.10 | 0.10 | 0.07 | 0.10 |  | 0.01 |
| *Microhyla taraiensis* | 0.07 | 0.11 | 0.10 | 0.11 | 0.10 | 0.05 | 0.11 | 0.07 |  |

Table S4: *Uncorrected p distance* within Group of Family Microhylidae

| *Microhyla ornata* | 0.002 | 0.001 |
| --- | --- | --- |
| *Microhyla chakrapanii* | n/c | n/c |
| *Microhyla fissipes* | n/c | n/c |
| *Microhyla mukhlesuri* 1 | n/c | n/c |
| *Microhyla mymensinghensis* | n/c | n/c |
| *Microhyla nilphamariensis* | 0.003 | 0.001 |
| *Microhyla mukhlesuri* 2 | 0.006 | 0.003 |
| *Microhyla rubra* | 0.002 | 0.002 |
| *Microhyla taraiensis* | 0.003 | 0.002 |

Table S5*: Uncorrected p distance* between Groups of Family Dicroglossidae

Table S6*: Uncorrected p distance* within Group of Family Dicroglossidae

| *Quasipaa shini* | n/c | n/c |
| --- | --- | --- |
| *Quasipaa boulengeri* | n/c | n/c |
| *Quasipaa jiulongensis* | n/c | n/c |
| *Quasipaa exilispinosa* | n/c | n/c |
| *Nanorana sp 1* | 0.000 | 0.000 |
| *Nanorana sp 2* | 0.000 | 0.000 |
| *Nanorana sp 3* | 0.008 | 0.004 |
| *Nanorana cf blanfordii* | 0.012 | 0.006 |
| *Nanorana cf ercepeae* | 0.000 | 0.000 |
| *Nanorana cf rostandi* | 0.007 | 0.007 |
| *Nanorana parkeri* | 0.009 | 0.005 |
| *Nanorana ventripunctata* | 0.000 | 0.000 |
| *Nanorana pleskei* | 0.002 | 0.002 |
| *Nanorana cf polunini* | 0.005 | 0.004 |
| *Nanorana liebigii* | 0.013 | 0.006 |
| *Nanorana yunnanensis* | n/c | n/c |
| *Nanorana taihangnica* | n/c | n/c |
| *Nanorana vicina* | 0.000 | 0.000 |
| *Allopaa hazarensis* | 0.000 | 0.000 |
| *Hoplobatrachus rugulosus* | 0.000 | 0.000 |
| *Hoplobatrachus tigerinus* 1 | 0.000 | 0.000 |
| *Hoplobatrachus tigerinus* 2 | 0.004 | 0.003 |
| *Euphlyctis ehrenbergi* | n/c | n/c |
| *Euphlyctis mudigere* | 0.000 | 0.000 |
| *Euphlyctis cyanophlyctis* | 0.000 | 0.000 |
| *Euphlyctis kalasgramensis* 1 | 0.007 | 0.004 |
| *Euphlyctis kalasgramensis* 2 | 0.003 | 0.003 |
| *Euphlyctis hexadactylus* | 0.021 | 0.009 |
| *Sphaerotheca pluvialis* | n/c | n/c |
| *Sphaerotheca dobsonii* | 0.005 | 0.005 |
| *Sphaerotheca breviceps* | 0.006 | 0.004 |
| *Sphaerotheca rolandae* | n/c | n/c |
| *Sphaerotheca pashchima* | 0.004 | 0.003 |
| *Fejervarya limnocharis* | 0.013 | 0.004 |
| *Menervarya refuscens* | n/c | n/c |
| *Minervarya greenei* | n/c | n/c |
| *Minervarya caperata* | n/c | n/c |
| *Minervarya asmati* | 0.042 | 0.017 |
| *Minervarya granosa* | 0.000 | 0.000 |
| *Minervarya syhadrensis* | 0.000 | 0.000 |
| *Minervarya pierrei* 2 | 0.001 | 0.001 |
| *Minervarya pierrei* 1 | 0.000 | 0.000 |
| *Minervarya kudremukhensis* | n/c | n/c |
| *Minervarya sahyadris* | n/c | n/c |
| *Fejervarya cancrivora* | 0.000 | 0.000 |
|  |  |  |
